# Sterol *O*-acyltransferase 2 chaperoned by apolipoprotein J facilitates hepatic lipid accumulation following viral and nutrient stresses

**DOI:** 10.1038/s42003-021-02093-2

**Published:** 2021-05-12

**Authors:** Hung-Yu Sun, Tzu-Ying Chen, Yu-Ching Tan, Chun-Hsiang Wang, Kung-Chia Young

**Affiliations:** 1 Department of Biomedical Engineering, College of Biology, Hunan University, Changsha, China; 2 Institute of Pathogen Biology and Immunology of College of Biology, Hunan Provincial Key Laboratory of Medical Virology, Hunan University, Changsha, China; 3 Institute of Molecular Medicine, College of Medicine, National Cheng Kung University, Tainan, Taiwan; 4 Department of Medical Laboratory Science and Biotechnology, College of Medicine, National Cheng Kung University, Tainan, Taiwan; 5 Division of Gastroenterology, Tainan Municipal Hospital, Tainan, Taiwan; 6 Institute of Basic Medicine, College of Medicine, National Cheng Kung University, Tainan, Taiwan; 7 Center of Infectious Disease and Signaling Research, College of Medicine, National Cheng Kung University, Tainan, Taiwan

**Keywords:** Mechanisms of disease, Non-alcoholic fatty liver disease

## Abstract

The risks of non-alcoholic fatty liver disease (NAFLD) include obese and non-obese stresses such as chronic hepatitis C virus (HCV) infection, but the regulatory determinants remain obscure. Apolipoprotein J (ApoJ) served as an ER-Golgi contact-site chaperone near lipid droplet (LD), facilitating HCV virion production. We hypothesized an interplay between hepatic ApoJ, cholesterol esterification and lipid deposit in response to NAFLD inducers. Exposures of HCV or free-fatty acids exhibited excess LDs along with increased ApoJ expression, whereas ApoJ silencing alleviated hepatic lipid accumulation. Both stresses could concomitantly disperse Golgi, induce closer ApoJ and sterol *O*-acyltransferase 2 (SOAT2) contacts via the N-terminal intrinsically disordered regions, and increase cholesteryl-ester. Furthermore, serum ApoJ correlated positively with cholesterol and low-density lipoprotein levels in normal glycaemic HCV patients, NAFLD patients and in mice with steatosis. Taken together, hepatic ApoJ might activate SOAT2 to supply cholesteryl-ester for lipid loads, thus providing a therapeutic target of stress-induced steatosis.

## Abstract (web summary)

Sun et al. show that hepatitis C virus or free-fatty acids increase lipid droplets and apolipoprotein J (ApoJ) expression while ApoJ silencing alleviates hepatic lipid accumulation. This study suggests that hepatic ApoJ may activate sterol *O*-acyltransferase 2 to supply cholesteryl-ester for lipid loads, thus presenting ApoJ as a promising target for treating stress-induced steatosis.

## Introduction

While the liver maintains body homoeostasis, excess lipid deposition accelerates the development of metabolic disorders and cardiovascular diseases^1^. Hepatic lipid overloads, referred to as steatosis, is a symptom of non-alcoholic fatty liver disease (NAFLD), occurring frequently not only in patients overweight and with obesity but also those of normal weight^2,3^. Multiple factors beyond the accumulation of extra energy in obesity facilitate lipid storage; for example, the aberrant intracellular lipid compositions might compromise hepatic functions, leading to an imbalanced metabolism^4,5^. Stress inducers, such as free fatty acids (FFAs) in a high-fat diet (HFD) or viral pathogenic factors in hepatitis C virus (HCV) infection, cause steatosis^4^.

Intracellular lipids are stored in lipid droplets (LDs), which consist of a surface coating of free cholesterol (Chol), phospholipids, and proteins surrounding neutral lipid esters in the core, and display highly dynamic structures^5^. LDs participate actively in the coordination of lipid metabolic events, providing working platforms to assemble the nucleocapsid of viral pathogens, such as HCV^6^ and dengue virus^7^. LD formation occurs from the budding of lipid ester globules covered by the outer leaflet membrane of the endoplasmic reticulum (ER). Subsequently, nascent droplets undergo expansion by LD mutual fusion or by local lipid ester synthesis and supply^8^. LDs contain a unique fatty core surrounded by a monolayer lipid membrane^5^, and communicate with other organelles at contact sites by being tethered to each other in close proximity^9,10^.

Storage lipid esters comprise triglyceride (TG) and cholesteryl ester (CE) at different patches in the core of LDs^5^. The enzymatic reactions are activated at ER–LD contact sites to supply TG biosynthesis for LD enlargement^11,12^; however, the induction of CE production remains to be investigated. Intracellularly, Chol provides building components to form organelles and plasma membranes, while CE, acylated from Chol by sterol *O*-acyltransferase (SOAT), has lower lipotoxicity than Chol, thereby constituting a more tolerant storage form^5^. Therefore, the catalysis of Chol esterification to CE might represent branching points from membranous system biosynthesis to lipid storage when cells have to shield toxic lipids.

Apolipoprotein J (ApoJ) is a Golgi-resident protein that serves as a stress-induced molecular chaperone^13,14^ in response to various cellular stimuli^15^. ApoJ could guide proper folding of client proteins, which might occur along with Golgi dispersion between organelles at contact sites or after secretion out of cells^13,16^, and is associated with very-low-density lipoproteins (VLDL)^17^. Production of infectious HCV virion was facilitated by ApoJ relocation from the perinuclear compact Golgi to the dispersed ER–Golgi contact sites surrounding LDs^16^. Coincidently, HCV infection depended on ER-resident SOAT-mediated Chol esterification leading to LD accumulation^18–20^. It is likely that organelle contact-site chaperones control Chol metabolism for microenvironmental guidance of SOAT enzymes^21^, which belong to a membrane-bound *O*-acyltransferase superfamily, when integral in hydrophobic transmembrane phase^22^.

In this study, we tested the hypothesis that hepatic ApoJ might modulate SOATs, facilitating CE production, and subsequently leading to abundant neutral lipid deposits in the LDs. Meanwhile, the clinical relevance of serum ApoJ to lipid parameters was examined in both chronic hepatitis C (CHC) and NAFLD patient groups, and in mice fed with HFD.

## Results

### Hepatic ApoJ participated in the induction of neutral lipid esters and LDs by HCV infection

**Fig. 1:**
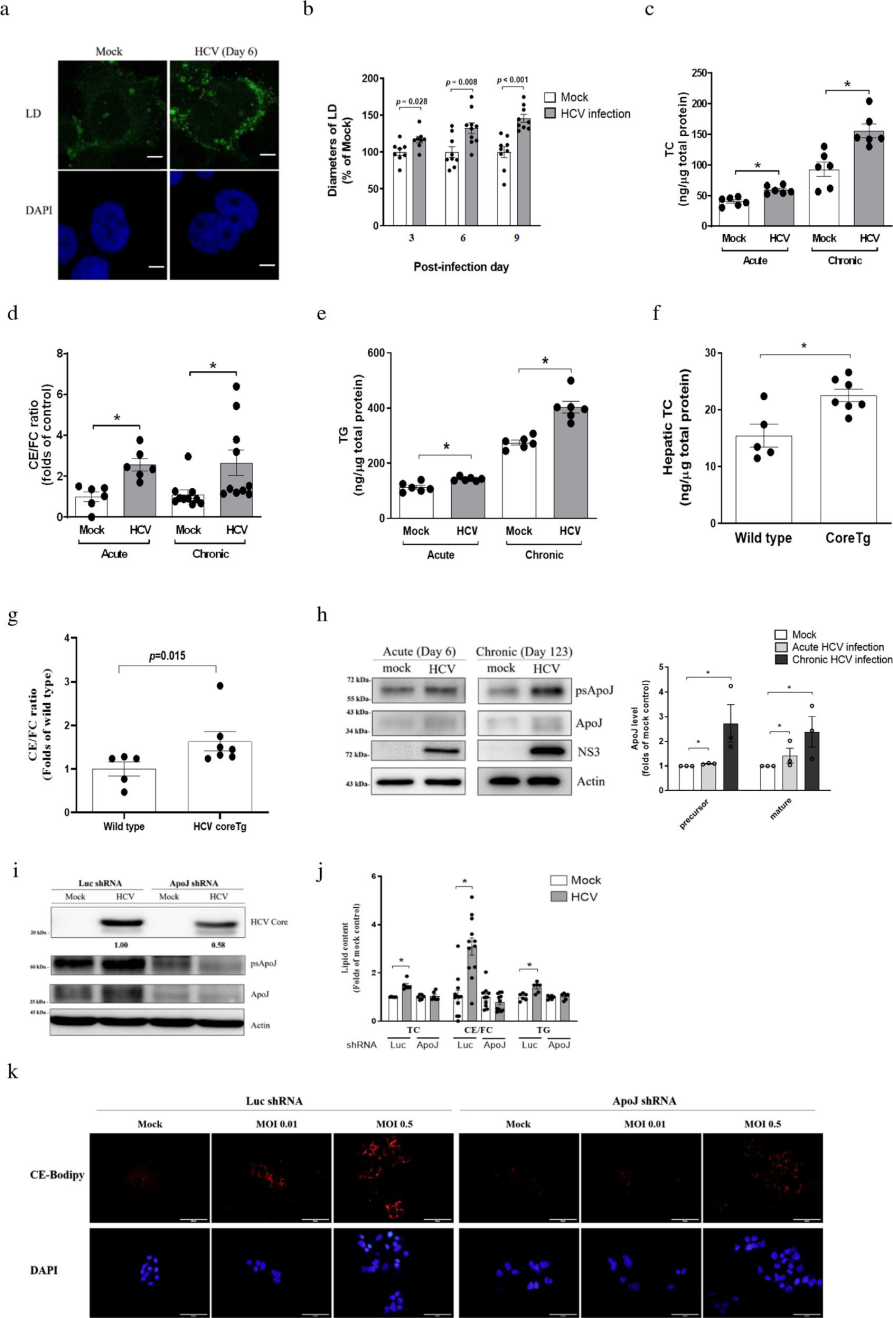
ApoJ participated in HCV-induced lipid accumulation. **a** Representative images of LDs in Huh7.5 cells with mock or HCV infection at day 6 post-infection. Scale bar, 5 μm. **b** The diameters of LDs (*n* > 2000) were quantified with ImageJ software. The data were collected from three independent experiments (*n* ≧ 8). Quantification of the intracellular contents of TC (**c**), CE/FC ratio (**d**), and TG (**e**) with acute or chronic HCV infections (*n* ≧ 6). The hepatic TC level (**f**) and CE/FC ratio (**g**) in wild type (*n* = 5) and HCV coreTg mice (*n* = 7). **h** Protein levels of ApoJ and HCV-NS3 in Huh7.5 cells with acute or chronic HCV infections. The right panel: ApoJ intensities (*n* = 3); psApoJ, ApoJ precursor. **i** Protein levels of ApoJ and HCV core in control and ApoJ-silenced Huh7.5 cells inoculated with HCV (MOI = 0.01) at day 6 post-infection. **j** TC, CE/FC ratio and TG contents and with HCV infection in the control and ApoJ-silenced Huh7.5 cells at day 6 post-infection. All results are presented as the mean ± SEM, and asterisks indicate statistical significance (*p* < 0.05). **k** Images of LDs visualized by CholEsteryl BODIPY™ 542/563 C_11_ tracer with MOI = 0.01 or 0.5 at post-infection day 3. Scale bar, 50 μm. Uncropped blots of **h** and thereafter are displayed in Supplementary Figs. 15–30.

HCV infection (MOI = 0.01) was undertaken to disturb lipid homoeostasis in Huh7.5 cells, as shown by the accumulation (Fig. 1a) and enlargement of LDs (Fig. 1b) at 3–9 days post-infection. For long-term changes, the lipids were analysed using cultivated reporter cells that mimicked the dynamics of hepatitis C viremia in patients^23^. The results showed the accumulative total Chol (TC), CE to free Chol (FC) ratio, and TG from acute to chronic stages of HCV infection (Fig. 1c–e). In HCV core transgenic (coreTg) mice fed with normal chow, hepatic TC (Fig. 1f) and CE/FC ratio (Fig. 1g) were increased significantly as early as 6 weeks old, while in comparison with that of the wild-type mice, serum TC (Supplementary Fig. 1a), serum TG (Supplementary Fig. 1b), and hepatic TG (Supplementary Fig. 1c) did not differ in the coreTg mice. Thus, the experiments supported that HCV infection could cause persistent hepatic enrichment of total neutral ester lipids, which might supply LDs for accumulation.

The host determinants facilitating the formation of hepatic neutral lipids and LDs were further investigated. HCV infection induced massive rearrangement of intracellular membranous components and inter-organelle contact sites for replicating viral genome^24^ and assembling the core-nucleocapsid^16^. Stress-induced ApoJ, functioning as an ER-Golgi-LD contact-site chaperone in assistance of infectious HCV virion production^16^, might concomitantly affect LD formation.

Herein, the effects of ApoJ on lipid accumulation were examined. Firstly, Huh7.5 cells with acute HCV infection had higher ApoJ levels (day 6) than did the mock control cells (Fig. 1h). In addition, the HCV protease, NS3, was persistently expressed in cells infected HCV for 123 days (Fig. 1h), indicating constitutively production of viral proteins in chronic HCV-infected cells^23^. Moreover, the intracellular ApoJ level was increased with chronic infection, confirming that both short and prolonged HCV infections could stimulate hepatic ApoJ expression. Next, compared to the controls, ApoJ-silenced cells (Supplementary Fig. 2) supported less efficient HCV infection at MOI = 0.01 for 6 days with a reduction of ~40% (Fig. 1i) and almost abolished the induction of TC, CE/FC ratio, and TG (Fig. 1j). With an attempt to test the effect of ApoJ knockdown on lipid levels without affecting viral spread and assembly, the experiments were also conducted with a high-viral titre at MOI = 0.5 for 3 days. Increment of TC, but not TG, occurred only in the cells expressing ApoJ (Supplementary Fig. 3a), while the HCV infections indicated by core protein expression levels were comparable in cells with and without ApoJ silencing (Supplementary Fig. 3b). Additionally, the amounts of CholEsteryl-BODIPY (CE-Bodipy)-labelled LDs were reduced in ApoJ-silenced cells with either low- (MOI = 0.01) or high-titre (MOI = 0.5) HCV infection at day 3 post-infection (Fig. 1k). Thus, ApoJ might contribute to HCV-induced lipid accumulation but with minimal effects on viral spreading since early stages of infection.

Focusing on its interplay with Chol, which is interchangeable in the forms of free sterol and steryl ester, we next investigated how ApoJ might affect hepatic lipid accumulation.

### HCV infection promoted the co-localization of ApoJ and SOAT proteins in the context of Golgi dispersion

**Fig. 2:**
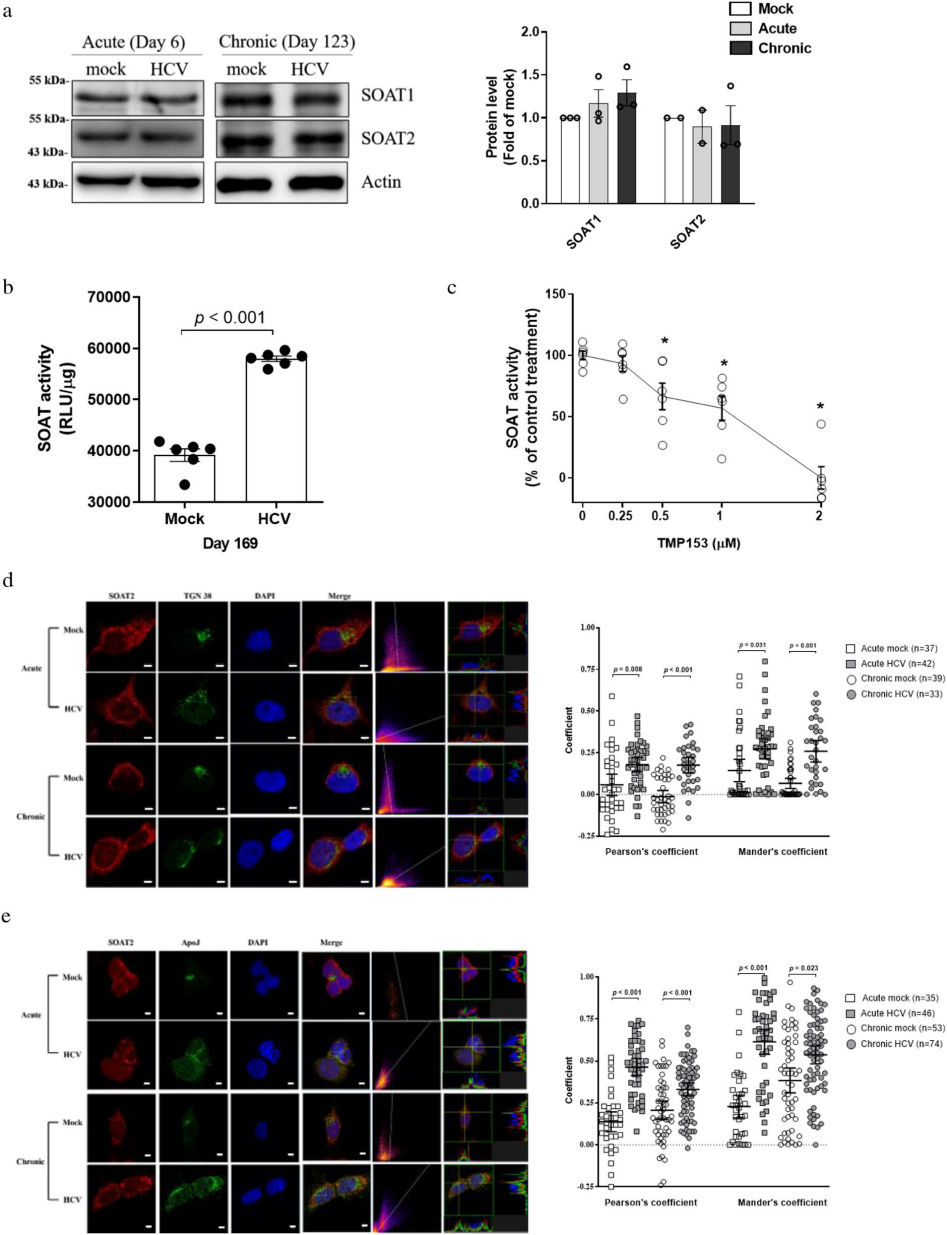
HCV infection induced the co-localization of ApoJ with SOAT2 and elevated SOAT activity in the context of Golgi dispersion. **a** Protein levels of SOAT1 and SOAT2 in Huh7.5 cells with acute or chronic HCV infections, with the intensities shown in the right panel (*n* ≧ 2). **b** HCV-infected Huh7.5 cells were fed with NBD-Chol, and the SOAT activity was evaluated (*n* = 6). **c** HCV-infected Huh7.5 cells were treated with 0.25–2 μM TMP153 for 16 h and the SOAT activity was quantified as in **b**. The asterisk indicates statistical significance (*p* < 0.05) as compared to the untreated control. **d** Representative IFA images of SOAT2 (red) and Golgi (TGN38, green) in HCV-infected cells. The scatter plots of red and green channels, as shown at the right of the merged image, and the Pearson’s and Mander’s colocalization coefficients were analysed by the Coloc2 plugin from ImageJ/Fiji (ImageJ-Fiji-ImgLib http://fiji.sc/), respectively, as shown in the right panel. The fluorescence intensity profile across the arrow for both channels was analysed by FVW31S software. **e** Representative IFA images of SOAT2 (red) and ApoJ (green) analysed as in **d**. Scale bar, 5 μm.

Unlike in adipocytes where the predominant lipid storage in the LD core is TG, hepatic LDs are prone to additionally storing CE, esterified from Chol by the SOAT enzymes. Upon examining both SOAT1 and SOAT2, neither acute nor chronic HCV infections had profound effects on their protein levels (Fig. 2a). However, the enzymatic activity was significantly increased in the infected cells (Fig. 2b), and treatment with TMP153, a pan inhibitor of SOATs, could confirm the specificity of the SOAT activity assay (Fig. 2c). Through co-localization analysis with calnexin, the results showed SOAT1 (Supplementary Fig. 4a) and SOAT2 (Supplementary Fig. 4b) resided predominantly in the ER and were unaltered by HCV infection. HCV infections could induce Golgi (TGN38) dispersion from perinuclear to cytoplasmic sites (Fig. 2d, the left panel). In parallel, as shown by the increased co-localization coefficients (*p* < 0.05) (Fig. 2d, the right panel), a significant portion of Golgi fragmentation occurred, thus leading to closer contacts with the SOAT2 than those in the mock controls. Meanwhile, the proportions of SOAT1 co-localized with the Golgi were comparable in the mock and HCV-infected cells (Supplementary Fig. 5a). Moreover, accompanying Golgi dispersion, HCV infection potentiated the intimate relationship prominently between ApoJ and SOAT2 with elevated co-localization coefficients (Fig. 2e, *p* < 0.05), whereas the co-localization levels of ApoJ with SOAT1 were not changed to the same extent (Supplementary Fig. 5b). The results suggested that HCV-induced stress might promote organelle contacts where the Golgi-resident ApoJ and the ER-resident SOAT2 were positioned adjacent to each other.

### Knockdown of ApoJ alleviated FFA-induced lipid accumulation

**Fig. 3:**
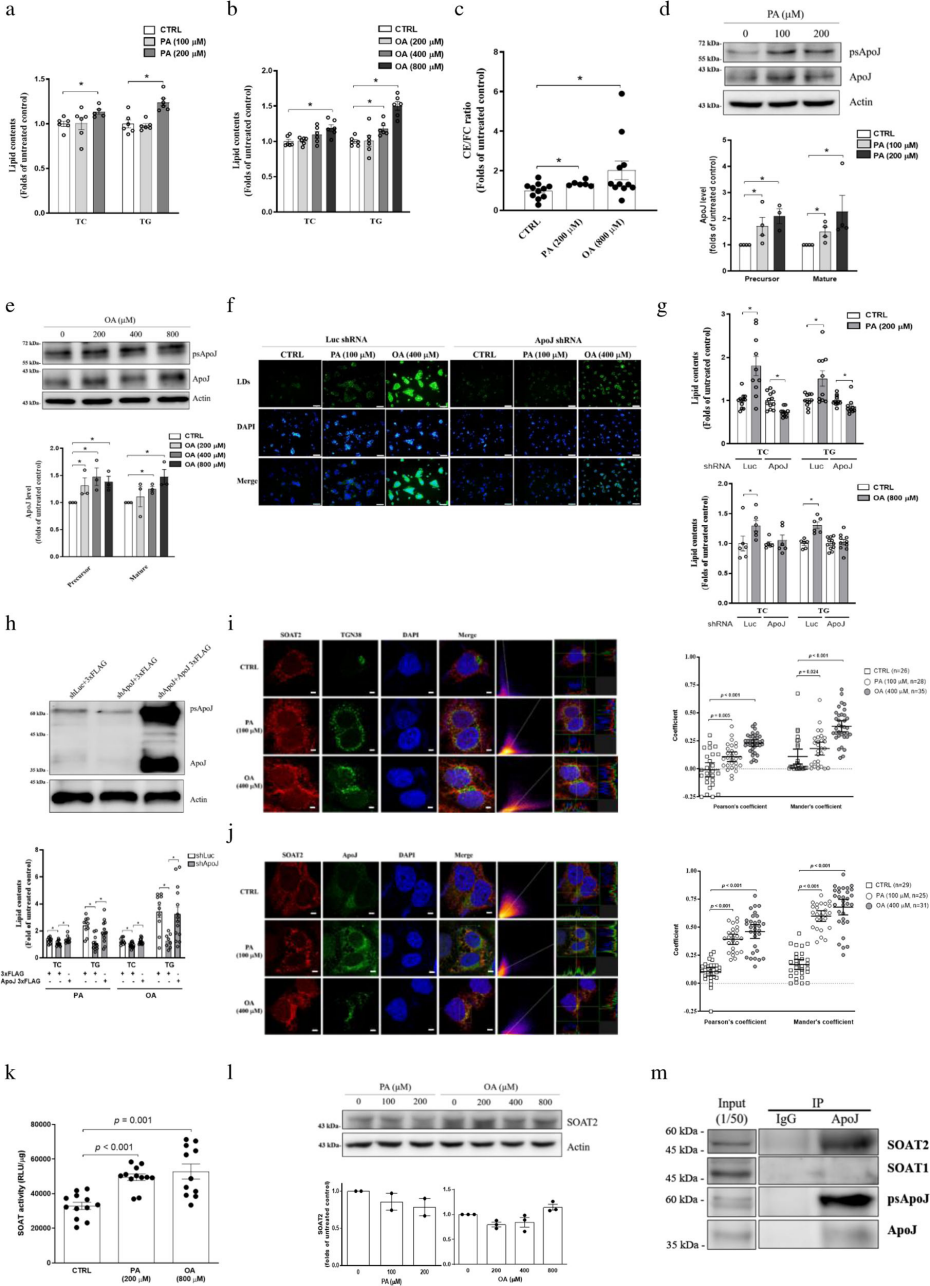
FFA-induced hepatic lipid accumulation depended on ApoJ. The cellular contents of TC and TG were quantified in Huh7 cells treated with PA (**a**) or OA (**b**) for 24 h (*n* ≧ 5), CE/FC ratio of PA and OA treated cells (**c**) (*n* ≧ 6), and ApoJ expression in Huh7 cells treated with PA (**d**) or OA (**e**). The lower panel: ApoJ intensities (*n* = 3); psApoJ, ApoJ precursor. **f** Representative images of LDs stained with BODIPY493/503, and **g** quantification of lipid contents in Huh7.5 cells bearing Luc or ApoJ shRNA treated with PA or OA for 24 h. Scale bar, 100 μm in **f**. **h** Quantification of lipid contents in the cells transfected with ApoJ expressing plasmid or control vector. All results are normalized to the respective untreated control and presented as the mean ± SEM (*n* ≧ 5), and asterisks indicate statistical significance (*p* < 0.05). Representative IFA images of SOAT2 (red) and Golgi (TGN38, green) (**i**); SOAT2 and ApoJ (green) (**j**) in PA- or OA-treated cells were analysed by Coloc2 plugin and FVW31S software as in Fig. 2d. Scale bar, 5 μm. Asterisks indicate statistical significance (*p* < 0.05). **k** The PA- (200 μM) or OA- (800 μM) treated Huh7 cells were fed with NBD-Chol, and the SOAT activity was evaluated and expressed as the mean ± SEM (*n* = 6). **l** The protein level of SOAT2 in Huh7 cells treated with PA or OA for 24 h. The lower panel: SOAT2 intensities (*n* ≧ 2). **m** Huh7 cells were treated with 800 μM OA for 24 h. The cell lysate was immunoprecipitated with antibody against ApoJ and examined by western blot analysis recognizing SOAT1, SOAT2, and ApoJ.

Alternatively, treatment with sub-lethal doses of FFAs (Supplementary Fig. 6) was exploited to induce hepatic lipid dysregulation. The intracellular lipid contents (Fig. 3a–c) and LDs (Supplementary Fig. 7) were increased dose-dependently with the administration of palmitic acid (PA) and oleic acid (OA) to Huh7 cells. The FFA-induced stress could also upregulate ApoJ levels (Fig. 3d, e), similar to that induced by HCV infection in Fig. 1h. Suppression of ApoJ expression reduced the FFA-mediated induction of LDs (Fig. 3f), TC and TG (Fig. 3g), which were reversible after the restoration of ApoJ expression (Fig. 3h).

### FFA treatment induced Golgi dispersion and increased SOAT/ApoJ co-localization and SOAT enzymatic activity

Similar to HCV infection, the FFAs barely affected the ER-resident distributions of either SOAT1 (Supplementary Fig. 8a) or SOAT2 (Supplementary Fig. 8b), but induced Golgi dispersed relocation (Fig. 3i) and promoted the co-localization of SOAT2/Golgi and SOAT2/ApoJ (Fig. 3i, j), as shown by the increased co-localization coefficients (*p* < 0.05). The co-localized SOAT1/Golgi (Supplementary Fig. 9a) and SOAT1/ApoJ (Supplementary Fig. 9b) remained constant with and without FFAs. FFAs significantly elevated the enzymatic activity of SOATs (Fig. 3k), with minimal changes in protein levels (Fig. 3l). Furthermore, the immunoprecipitation assay also confirmed that ApoJ might interact with SOAT2, but not SOAT1 (Fig. 3m). Taken together, the results suggested that Golgi dispersion and an increase in SOAT2/ApoJ co-localization and SOAT activity might occur by either HCV infection or FFA exposure as stress inducers in the development of hepatosteatosis.

### ApoJ participated in the enhancement of SOAT2 enzymatic activity

**Fig. 4:**
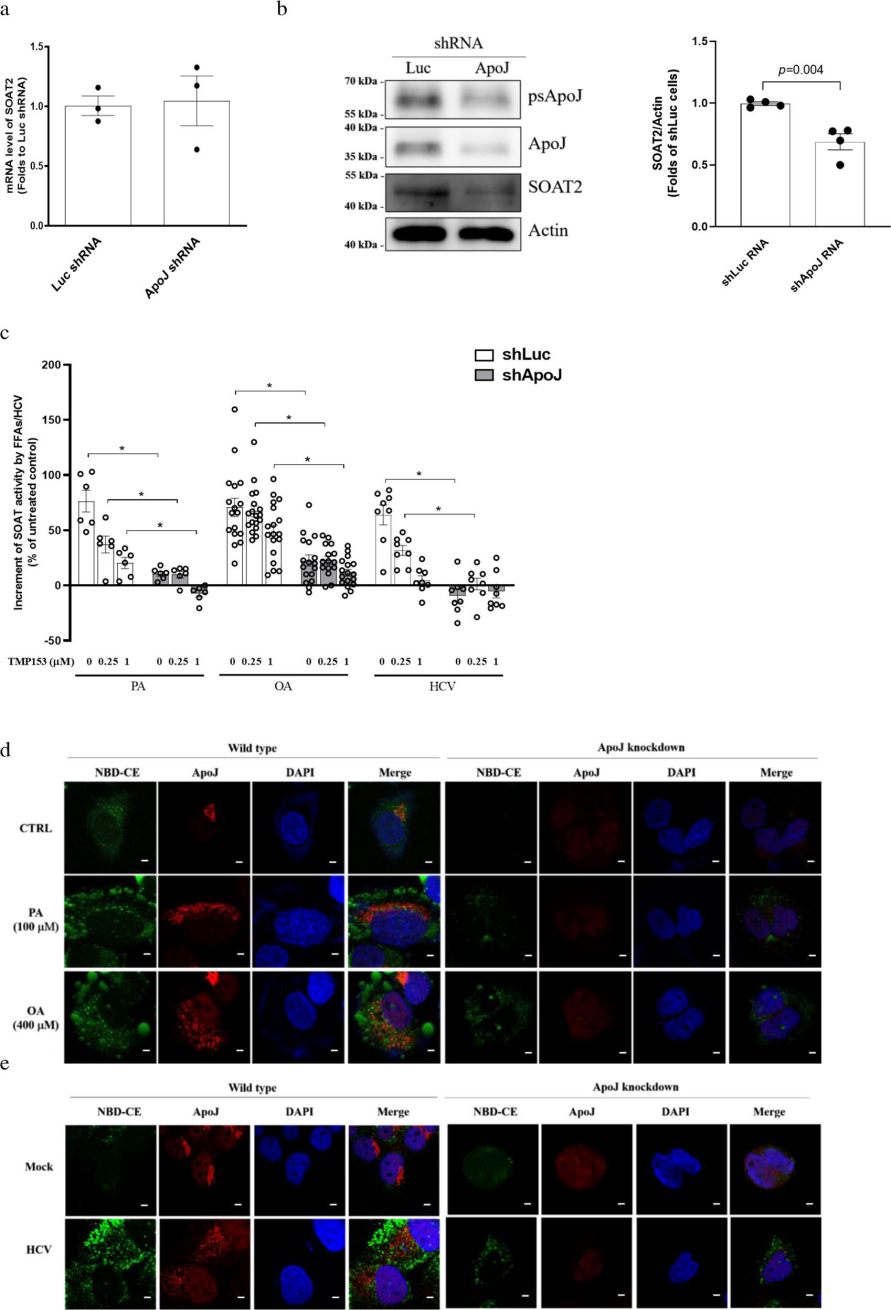
ApoJ silencing reduced SOAT2 expression and SOAT enzymatic activity. Huh7.5 cells bearing Luc or ApoJ shRNA were established to examine a SOAT2 mRNA (*n* = 3); **b** SOAT2 protein expression (representative image in left panel; SOAT2 intensity in right panel, *n* = 4) and; **c** SOAT enzymatic activity in the presence of FFAs or HCV infection at MOI = 0.01 for 6 days with and without TMP153 treatment; and **d**, **e** image visualization of NBD-CE under confocal microscopy observation. The data were presented as the mean ± SEM (*n* ≧ 6), and asterisks indicate statistical significance (*p* < 0.05) between the control and ApoJ knockdown cells. Scale bar, 5 μm.

In the ApoJ knockdown cells, the SOAT2 mRNA (Fig. 4a) was comparable to that in the control cells, whereas protein level (Fig. 4b) was downregulated. Both FFAs and HCV could enhance the SOAT activity by approximately 70% of that of their corresponding control counterparts, but by less than 20% in the corresponding ApoJ knockdown cells (Fig. 4c). Furthermore, ApoJ-silencing is consistently accompanied by a reduction in the fluorescent spots of NBD-CE induced by FFA treatment (Fig. 4d) or HCV infection (Fig. 4e).

### SOAT2 intrinsically disordered regions (IDRs) were required for FFA-induced co-localization with ApoJ

**Fig. 5:**
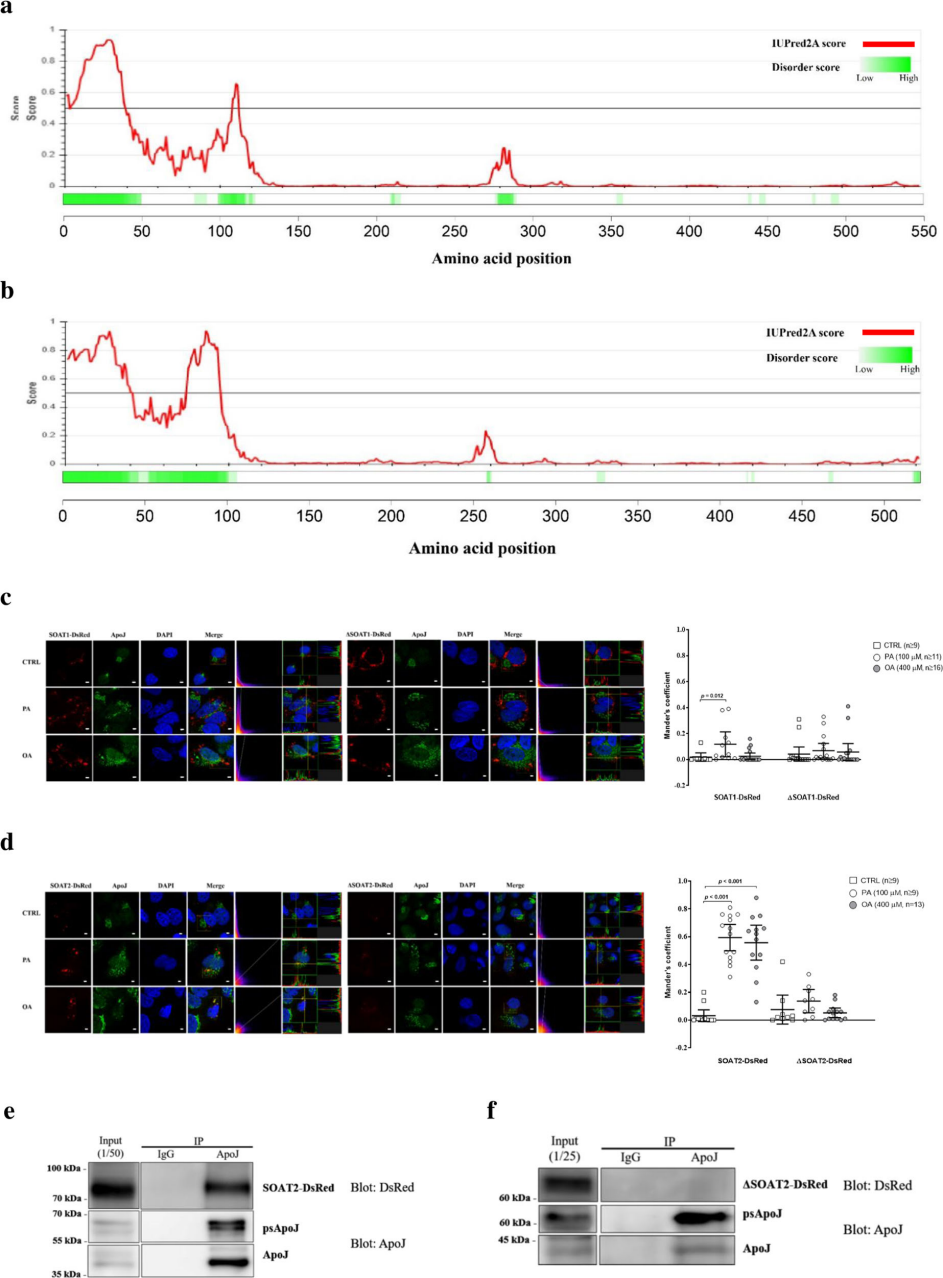
ApoJ co-localized with SOAT2 by recognizing the IDR region. The IDR regions of SOAT1 (UniProt ID: P35610, **a**) and SOAT2 (UniProt ID: O75908, **b**) were predicted by D^2^P^2^ (http://d2p2.pro/search) and IUPred2A (https://iupred2a.elte.hu/) algorithms, respectively. Huh7 cells were transfected with SOAT1-DsRed (**c**, the left panels), IDR-truncated SOAT1-DsRed (ΔSOAT1-DsRed, **c**, the right panels); SOAT2-DsRed (**d**, the left panels), and IDR-truncated SOAT2-DsRed (ΔSOAT2-DsRed, **d** the right panels), followed by administration of PA (100 μM) or OA (400 μM) for 24 h, and visualized with confocal microscopy. The fluorescence intensity was analysed by FVW31S software. The Mander’s coefficient was analysed by ImageJ with the Coloc2 plugin from ImageJ/Fiji shown above. Scale bar, 5 μm. Huh7 cells were transfected with plasmids encoding SOAT2-DsRed (**e**) or ΔSOAT2-DsRed (**f**). The cell lysates were immunoprecipitated with antibody against ApoJ and examined by western blot analysis recognizing DsRed and apoJ.

IDRs refer to protein segments lacking stable tertiary structures; they exist abundantly in signalling processes as structurally flexible and diverse ensembles^25^. Dual software predictions with amino acid sequencing showed that the 1st–38th residues of SOAT1 (Fig. 5a) and the 1st–41st residues of SOAT2 (Fig. 5b) might contain IDRs, but the disordered regions of ApoJ were scattered and less prominent (Supplementary Fig. 10). To investigate whether the SOAT proteins might undergo IDR-facilitated interaction with ApoJ, the recombinant SOAT1, SOAT2, and the corresponding ΔSOAT1 and ΔSOAT2 IDR deletion mutants tagged with DsRed were constructed (Supplementary Fig. 11). Though PA slightly increased the interaction of ApoJ and SOAT1, the effect of FFA treatment on interaction of ApoJ with SOAT1 or ΔSOAT1 was minimal (Fig. 5c). FFA promoted the co-localization of SOAT2 with ApoJ, but not that of ΔSOAT2 (Fig. 5d). Furthermore, the interaction between ApoJ and SOAT2 was abolished in ΔSOAT2 IDR deletion mutants (Fig. 5e, f).

### Circulating ApoJ correlated positively with TC and low-density lipoprotein (LDL) levels in both patient groups with CHC and NAFLD, and in mice with high-fat diet (HFD)-induced steatosis

**Fig. 6:**
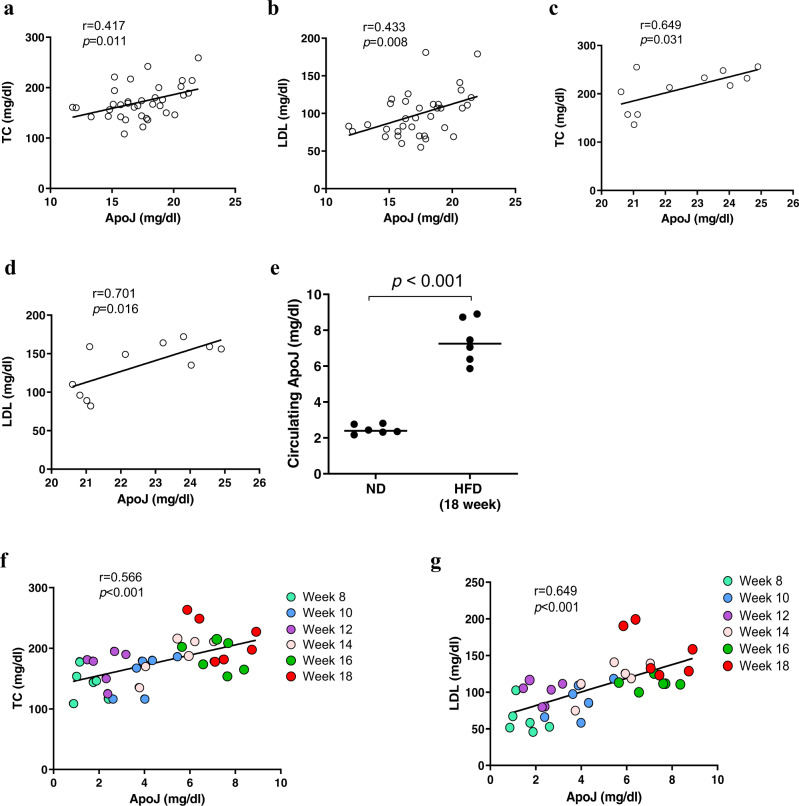
The circulating ApoJ levels exhibited positive correlations with LDL and Chol. The correlations of ApoJ vs. **a** TC and **b** LDL in CHC patients; and **c** TC and **d** LDL in NAFLD patients with steatosis grade ≧ 2 are shown. **e** The serum ApoJ levels of mice with an ND or HFD for 18 weeks were determined by ELISA quantification and are presented as the mean ± range (*n* = 6). The correlations of serum ApoJ vs. TC (**f**) and LDL (**g**) in mice with HFD-induced steatosis from 8 to 18 weeks feeding time intervals.

The CE synthesis catalysed by SOAT2 supplies both neutral lipid cores for cytosolic LD storage as well as secretory lipoproteins^26^. Since ApoJ is released from hepatocytes as B-100-containing lipoprotein residents, the association of circulating ApoJ in blood lipid components was then examined. Consistent with our previously study, serum ApoJ level correlated positively with HCV RNA titre (*r* = 0.340; *p* = 0.042, Table 1)^16^. The results further revealed positive correlations between serum ApoJ/TC and ApoJ/LDL in patients with CHC (*r* = 0.417, 0.433; *p* = 0.011, 0.008, Fig. 6a, b) and in patients with NAFLD with steatosis grade ≧ 2 (*r* = 0.649, 0.701; *p* = 0.031, 0.016, Fig. 6c, d). Meanwhile, serum ApoJ showed no correlations with high-density lipoprotein (HDL) and TG levels (Table 1).

**Table 1:** The correlation between circulating ApoJ and analytical components in CHC and NAFLD patient groups.

	CHC (*n* = 36)	NAFLD (*n* = 41)	
		Steatosis grade = 1 (*n* = 30)	Steatosis grade ≧ 2 (*n* = 11)
Age (years)	−0.226 (0.185)	0.317 (0.088)	0.064 (0.853)
BMI (kg/m^2^)	0.056 (0.745)	−0.351 (0.057)	−0.309 (0.356)
HCV RNA (IU/mL)	0.340 (0.042)	Not detectable	Not detectable
GOT (U/L)	−0.195 (0.254)	0.032 (0.866)	0.434 (0.182)
GPT (U/L)	−0.062 (0.715)	0.142 (0.456)	0.406 (0.215)
TC (mg/dL)	0.417 (0.011)	0.252 (0.179)	0.649 (0.031)
TG (mg/dL)	0.059 (0.731)	0.221 (0.240)	0.059 (0.864)
LDL (mg/dL)	0.433 (0.008)	0.057 (0.767)	0.701 (0.016)
HDL (mg/dL)	0.043 (0.802)	0.355 (0.054)	0.384 (0.244)

Results by Pearson’s correlation coefficients (*p* value).

Next, we analysed the dynamic changes in serum lipid parameters and ApoJ in HFD-induced steatosis in mice. The results showed that HFD treatment induced hepatic TC (Supplementary Fig. 12a), TG (Supplementary Fig. 12b), and CE/FC ratio (Supplementary Fig. 12c) accumulation and increased serum TC, TG, LDL, and HDL levels (Supplementary Fig. 13a–d), as well as increased levels of hepatic (Supplementary Fig. 12d) and serum ApoJ (Fig. 6e and Supplementary Fig. 13e). Consistently, serum ApoJ correlated positively with TC and LDL levels in HFD mice (*r* = 0.566, 0.649; *p* < 0.001, Fig. 6f, g) but not in mice fed with normal diet (ND) (Supplementary Fig. 14). Mice serum ApoJ showed no correlations with HDL and TG levels (Supplementary Table 1). Together, the results suggested that ApoJ might facilitate the supply of CE for B-100-containing lipoprotein formation and escort their release into circulation as a serological marker for stress-induced steatosis.

## Discussion

Disturbance of hepatic lipid homoeostasis causes aberrant intracellular accumulation that predisposes individuals to NAFLD, cardiovascular, and systemic metabolic diseases. Cellular neutral lipids are primarily stored in the LD core, of which the outer phospholipid monolayer coat forms contact sites with other organelles in close proximity^5,8^. The intimate interactions between LDs, ER, and the Golgi at contact sites might facilitate infectious HCV virion production^16^ and also contribute to hepatic manifestation of lipid accumulation (the present study). Our results suggested that intracellular ApoJ-mediated SOAT activation might respond to stress inducers originating from either obesity, such as FFAs, or non-obesity, such as HCV infection, and provide CE for hepatic LD enlargement. The results further showed high serum ApoJ concentration in patients with metabolic steatosis and HFD-induced steatosis mice, and positive correlations between serum ApoJ and atherogenic lipid profiles (Fig. 6c, d, f, g); this suggests that ApoJ-induced CE production might supply not only intracellular lipid overloads but also secretory lipoproteins acting as vesicles of ApoJ delivery into the bloodstream. In agreement with the results that CHC patients exhibited clinical manifestations similar to those in the overweight-induced progression of NAFLD^4^, the present study suggested that CHC or NAFLD patients might have a similar regulatory scenario in which circulating ApoJ correlated positively with TC and LDL (Fig. 6).

In the human liver, the lipid contents must be no more than 5% of the total mass; otherwise, hepatic steatosis develops^27^. In mature adipocytes, where the LD core consists of TG 10-fold higher than in other tissues, the expression of SOATs is low^28^, whereas the hepatocytes, possessing active SOAT enzymes, store both TG and CE in the LD and secrete abundant CE in forming lipoproteins^20^. Thus, ApoJ/SOAT-mediated CE-containing LD formation might contribute to the regulation of lipid accumulation in the liver but does not necessarily apply to the mature adipose tissue. Alternatively, the SOAT activities in preadipocytes might regulate SREBP1-mediated gene activation for de novo TG synthesis during adipogenesis^29^. Nevertheless, whether and how ApoJ acts as a stress-induced molecular chaperone, possibly regulating the activities of enzymes for TG synthesis, is worthy of further investigation.

The ApoJ protein, synthesized as a precursor, is cleaved into alpha- and beta-chains by furin-like proprotein convertase, and regulates cellular functions in various tissue types by both intra- and extra-cellular directed signals^30^, involved in aging, neoplasms, diabetes, kidney disease, and late-onset Alzheimer’s disease^31–33^. ApoJ, belonging to the apoE family, is abundant in lipoproteins^17,34^ and acts as an extracellular chaperone that guides refolding of client proteins^34^. The blood B-100-tagged lipoproteins are secreted from the liver as VLDL, which first assembles as TG- and CE-containing particles, and then converts into LDL via TG hydrolysis by lipoprotein lipase. Distinctly, the HDL particle is made of hepatic-derived lipid-free apolipoprotein AI which can efflux FC from peripheral tissue for reverse transport back to the liver. The results of our current study and Aronis et al.^35^ showed positive correlations between serum ApoJ and LDL, but not with HDL, suggesting that ApoJ might participate in the hepatic CE loading of TG-rich lipoproteins, but barely in the peripheral FC efflux for HDL assembly. In addition, the amphipathic ApoJ might mediate protein–lipid interplay in control of the exchange events between various circulating lipoprotein classes and those occurring at the various organellar contact sites^16^. Previous studies showed that ApoJ could evade the secretion pathway and reach the cytosol in the presence of ER stressors^36^, or move to the mitochondria by apoptosis inducers^37^. Thus, it is reasonable that the redistribution of ApoJ under stress conditions might facilitate the interaction with cytoplasmic domain of SOAT2. As shown in Fig. 5d, ApoJ might facilitate the formation of large puncta of SOAT2 via the interaction with IDRs, implying the involvement of phase-separation-based condensation as a membraneless compartment for sensing and reaction^38^. Furthermore, ApoJ silencing reduced the protein level of SOAT2 (Fig. 4b); however, the intracellular SOAT2 was not increased significantly with HCV infection (Fig. 2a) or FFA treatment (Fig. 3l) when induction of ApoJ occurred in those cells (Figs. 1h and 3d). Combined our results which suggested that ApoJ binding could stabilize SOAT2 and the image data (Figs. 2e and 3j) showing not all SOAT2 proteins co-localized with ApoJ, it was possible that the degradation of non-ApoJ bound SOAT2 might be accelerated with stimuli, leading to comparable levels of SOAT2 in the stress-induced cells as the corresponding mock infected (Fig. 2a) or un-treated (Fig. 3l) controls.

Recently, the association of ApoJ with metabolic diseases was identified. FFAs might activate the secretion of ApoJ from adipocytes and impair hepatic insulin sensitivity via the surface LRP2 receptor, megalin^39^. On the other hand, the exogenous overexpression of ApoJ might down-regulate hepatic lipogenesis via the repression of SREBP-1c transcription, and the trans-activation activities of liver X receptors and specificity protein 1^40^, however, SREBP-1 and liver X receptors might up-regulate ApoJ transcription^41^. Thus, ApoJ expression is likely under multifactorial control. In the present study, both HCV and FFAs could induce ApoJ-mediated SOAT catalytic efficiency near the ER-Golgi-LD contact site and therefore facilitate CE production and LD accumulation. It was proposed that ApoJ might function as an inter-organellar molecular chaperone thus maintaining proper folding of proteins, such as SOAT. Notably, the lipid loads were almost unchanged in the untreated cells with and without ApoJ, but accumulated only in the ApoJ-positive hepatocytes with FFA treatment or HCV infection, suggesting that ApoJ regulation on lipids were exerted merely in response to various cytotoxic insults. The serum ApoJ levels exhibited positive correlations with blood TC and LDL (Fig. 6) and glucose^16^, as well as elevation in patients with obesity^39^, type 2 diabetes^42,43^, cardiovascular diseases^35,39,44,45^, and in mice models of hepatosteatosis and non-alcoholic steatohepatitis^39^. Since excess lipids are toxic, the strategy for suppression of ApoJ and subsequent reduction of LDs might be translated into alleviation of cellular stress levels in hepatocytes under steatosis inducers. Taken together, the studies suggested a combined working model wherein extracellular ApoJ could induce liver fat accumulation via signalling through megalin^39^, while intracellular ApoJ could increase CE and LDs mediated by SOAT activity.

**Fig. 7:**
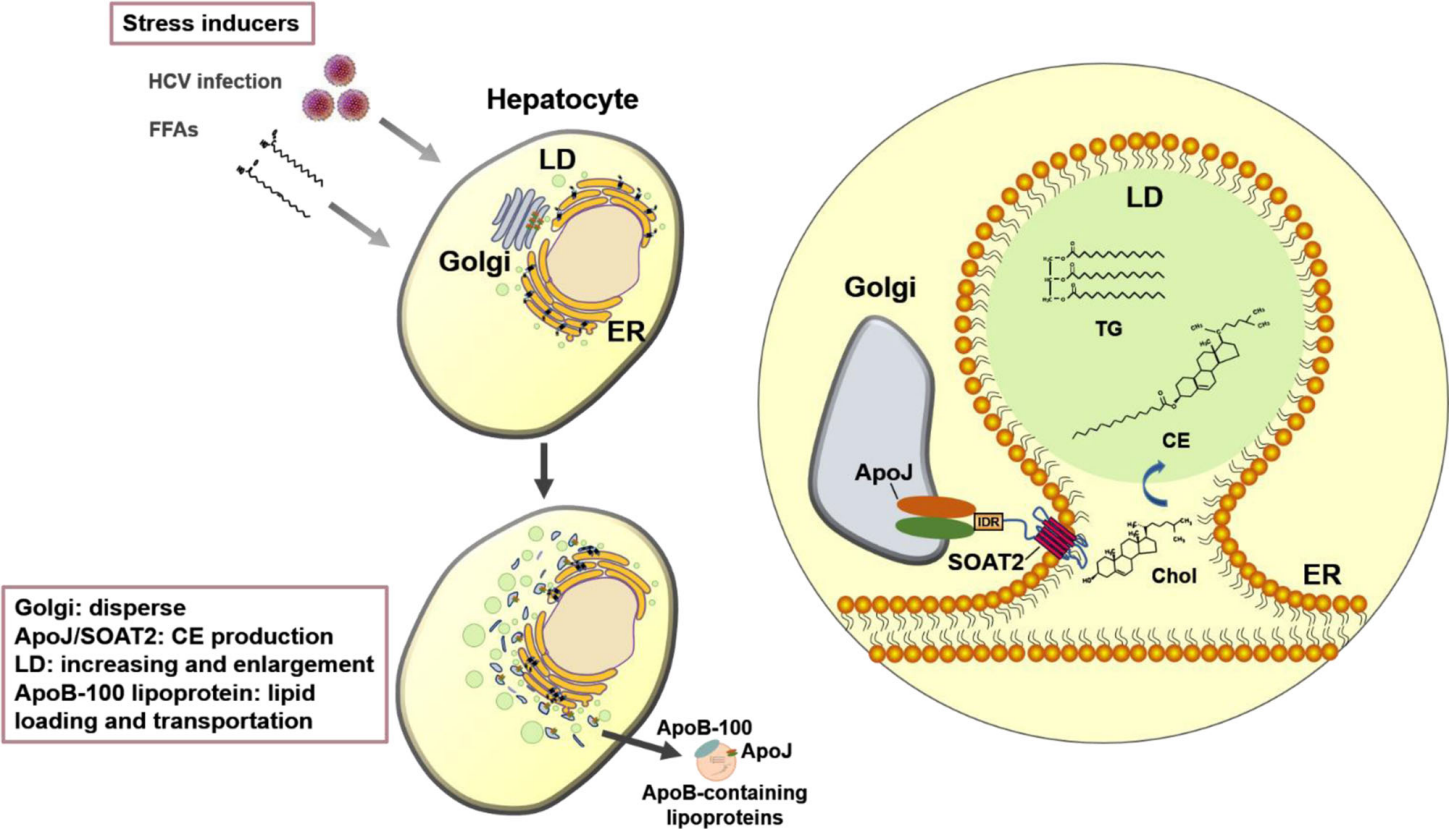
The proposed model of ApoJ/SOAT2 axis in stress-induced hepatosteatosis. In hepatocytes facing stress, ApoJ mobilizes along with dispersed Golgi to the Golgi–ER contact site, where ApoJ interacts with ER-resident SOAT2 at the N-terminal IDR region and coordinates Chol esterification to produce CE for LD deposition and lipoprotein loading.

In conclusion, hepatic ApoJ might interact with SOAT2 through IDR region, facilitating Chol esterification for LD deposition and lipoprotein loading under steatosis inducers (Fig. 7).

## Methods

### Antibodies

Monoclonal antibodies recognizing HCV core (ab2740) and NS3 (ab65407) were purchased from Abcam (Cambridge, UK); ApoJ (ARG62961) for IFA from Arigo Biolaboratories (Taipei, Taiwan); actin (MAB1501) from Millipore (Billerica, MA); and DsRed (tcba13674) from Taiclone Biotech Corp. (Taipei, Taiwan). Polyclonal antibodies recognizing human ApoJ for western blot (WB) analysis (ab69644) was purchased from Abcam; human ApoJ (sc-6419) for immunoprecipitation from Santa Cruz Biotechnology (Santa Cruz, CA); mouse ApoJ for WB analysis (PA5-46931) from Thermo Fisher Scientific Inc. (Waltham, MA); SOAT1 (ARG56476) and SOAT2 (ARG57814) for WB analysis from Arigo Biolaboratories; and SOAT 1 (bs-7544R) and SOAT 2 (bs-5020R) for IFA from Bioss Antibodies (Beijing, China). Goat anti-mouse Alexa-488-, and anti-rabbit Alexa-568-conjugated secondary antibodies were purchased from Thermo Fisher Scientific Inc.; Goat anti-mouse HRP- and anti-rabbit HRP-conjugated secondary antibodies were purchased from Chamot Biotechnology Co. Ltd (Shanghai, China).

### Patient samples

The patient samples included 36 CHC without other forms of viral hepatitis and liver diseases, and 41 NAFLD without alcohol consumption (<20 g/day), hepatitis B or C virus infections and other forms of liver diseases (Table 2). Since high glucose concentrations stimulate ApoJ transcription^46^ and elevate serum ApoJ^16^, the patients were recruited by a cut-off of less than 6% of the normal haemoglobin A1c (HbA1c) range to avoid confounding effects of glucose.

**Table 2:** Patient demographics of the enrolled CHC and NAFLD patient groups in the study.

	CHC (*n* = 36)	NAFLD (*n* = 41)		Statistics between groups^*^ *p* value < 0.05
		Steatosis grade = 1	Steatosis grade ≧ 2	
Gender (F/M)	24/12	13/17	4/7	
Age (years)	66 (46–81)	50 (25–85)	54 (27–72)	a, b
BMI (kg/m^2^)	24.8 (18.7–34.8)	25.3 (21.1–32.5)	27.8 (24.1–36.7)	b, c
Body fat (%)	35.7 (20.7–51.7)	29.2 (18.4–44.1)	33.8 (20.6–44.6)	a
GOT (U/L)	56 (23–346)	21 (15–113)	26 (15–95)	a, b
GPT (U/L)	74 (19–349)	27 (13–118)	46 (15–177)	a, b, c
TC (mg/dL)	167 (108–259)	198 (149–312)	217 (136–256)	a, b
TG (mg/dL)	85 (36–176)	127 (30–338)	166 (49–513)	a, b
LDL (mg/dL)	95 (55–181)	120 (78–240)	149 (82–172)	a, b
HDL (mg/dL)	59 (26–90)	51 (30–74)	49 (26–77)	a
HbA1c (%)	5.4 (4.7–5.9)	5.7 (4.2–5.9)	5.6 (5.1–5.9)	a, b
Plasma apoJ (mg/dL)	17.4 (11.8–22.0)	22.5 (15.8–26.8)	22.1 (20.6–24.9)	a, b
HCV RNA (Log10 IU/mL)	6.0 (2.6–6.9)	Not detectable	Not detectable	

Results by median (range).

^*^Statistics between groups: a, CHC vs. steatosis grade = 1; b, CHC vs. steatosis grade ≧ 2 and c, steatosis grade = 1 vs. grade ≧ 2 were evaluated by Mann–Whitney *U* test.

The patients were recruited from Tainan Municipal Hospital, Taiwan, with the approval of the local Institutional Review Board and each participant signed an informed consent form.

### Mouse samples

Six-week-old C57BL/6 mice (Scientific Integration Design Service Corporation, Taiwan) were induced to be obese by feeding an HFD (60% fat) for 18 weeks, while the controls were fed an ND (4–5% fat). The transgenic mice model with a hepatic expressing genotype-1b HCV core was previously generated^47^. The serum and liver samples were harvested and stored at −80 °C until laboratory analysis.

This study was approved by the local Animal Research Committee, and all experimental procedures followed the guidelines of the Public Health Service policy on Humane Care and Use of Laboratory Animals.

### Cell culture

Huh7.5 cells for HCV infection and Huh7 cells for FFA treatment^47^ were cultured in Dulbecco’s modified Eagle’s medium (HyClone, South Logan, UT) supplemented with 10% FBS and 1% penicillin/streptomycin; those (Huh7.5 with secreted alkaline phosphatase reporter gene, Huh7.5-SEAP) bearing acute and chronic HCV infections were additionally supplemented with 2 μg/mL blasticidin^23,48^; and those bearing Luc or ApoJ shRNA were supplemented with 2 μg/mL puromycin^16^. For FFA-treated cells, PA stock reagent was dissolved in alcohol and OA in DMSO, and then diluted to the indicated concentrations by DMEM.

### Establishment of acute and chronic HCV infections

Huh7.5 and Huh7.5-SEAP cells were inoculated with HCVcc at low (0.01) to high (0.5) MOI, grown for a couple of days to establish acute infection^48^, and then a part of infected Huh7.5-SEAP cells at low-MOI was sub-cultured every 7 days for a long-term period over 3 months to establish chronic infection^23^. For HCV-infected cells, the NS3/4A protease-based SEAP reporter activity was continuously measured in harvested medium every other day that ensured active cell function in support of HCV chronicity^23^. The expression of viral proteins was examined by WB analysis.

### Plasmid construction

The coding region of ApoJ was cloned into p3XFLAG-CMV-14^16^; the coding regions of SOAT1, ΔSOAT1 (deletion of residues 1-38), SOAT2, and ΔSOAT2 (deletion of residues 1-41) were obtained by the gene synthesis method and cloned into the pDsRed2-Mito expression vector (Leadgene Biomedical, Inc.).

### Immunofluorescence stain (IFA)

Immunofluorescence assays were performed as follows^16^. Briefly, the indicated cells were fixed with 4% paraformaldehyde, permeabilized with 0.2% Triton X-100, blocked with 2.5% bovine serum albumin, reacted with specific primary antibodies and visualized with Alexa Fluor 488-, or 568-conjugated secondary antibodies as appropriate. BODIPY493/503 or CholEsteryl BODIPY™ 542/563 C_11_ (Thermo Fisher Scientific Inc.) was applied for tracing LDs. The images were captured using a multi-photon laser scanning microscope (FluoView^TM^, Olympus, Tokyo, Japan).

Pearson’s correlation and Mander’s overlap coefficients were used as statistics for quantifying co-localization of target proteins in region of interest with Coloc2 plugin from ImageJ/Fiji software^49^, the former by assessing the linear relationship of fluorescence intensities between the two images, and the latter by counting the fraction of pixels with co-occurrence of the two fluorescent images which were primarily insensitive to the signal intensities. The fluorescence intensity profile across the arrow for both green and red channels was analysed using Olympus FV31S-SW software (Olympus, Japan).

### WB analysis

Cell lysates were separated by sodium dodecyl sulfate-polyacrylamide gel electrophoresis, transferred to a polyvinylidene fluoride membrane, incubated with specific primary antibodies followed by horseradish peroxidase-conjugated secondary antibodies, and developed with Western Lighting (Perkin-Elmer Life Sciences, Waltham, MA). Chemiluminescent signals were quantified by AlphaImage 2200 software (Lab Recyclers, Gaithersburg, MD).

### Immunoprecipitation assay

The Huh7 cells were seeded on 10-cm dishes at a density of 3 × 10^6^/dish, treated with 800 μM OA for 24 h to examine endogenous proteins, or transfected with the expression plasmids encoding SOAT2-DsRED or ΔSOAT2-DsRED (4 μg of each plasmid) for 48 h, and lysed by NETN lysis buffer (150 mM NaCl, 20 mM Tris-base, 5% NP-40, and 1 mM EDTA, pH 8.0) supplemented with proteinase inhibitor cocktail (Merck, MA). Cell lysate (4 mg of total protein) was pre-cleaned with Protein G sepharose (Croyez Bioscience, Tainan, Taiwan), immunoprecipitated with 2 μg of antibody against ApoJ or goat IgG (Merck) at 4 °C overnight, washed by ice-cold NETN lysis buffer, and examined by WB analysis recognizing ApoJ, SOAT1, SOAT2, and DsRed.

### Quantification of lipids, lipoproteins, and ApoJ

Total lipids were extracted with hexane:isopropanol (3:2; v/v) from the cultivated cells^47^. Liver tissues were homogenized in PBS using a MagNA lyser (Roche Diagnostics, Germany) and solubilized by 0.5% deoxycholate^50^. TC, CE, FC, and TG were quantified respectively with Infinity reagents (Thermo Fisher Scientific Inc.) and Total Cholesterol Assay Kits (Cell Biolabs, Inc., CA), normalized with total protein. Serum TC, TG, and HDL levels in mice were quantified by the corresponding kits purchased from Fortress Diagnostics (Antrim Northern Ireland, UK). The serum LDL level of mice was calculated by Friedewald’s formula. The circulating ApoJ was determined using with ELISA (Clusterin Quantikine kit, R&D Systems, Minneapolis, MN)^16^.

### ApoJ silencing

Huh7.5 cells were inoculated with VSV-G pseudotyped lentivirus expressing shRNA against ApoJ (TRCN0000078610) or luciferase control (TRCN0000072247) purchased from the Clinical Medicine Research Center, National Cheng-Kung University, Taiwan. The polyclonal cells were selected and stably maintained with puromycin. The target sequences of shRNA are shown in Supplementary Table 2.

### MTS assay

Cells were exposed to FFAs at the indicated doses for 24 h, and the cell viabilities were estimated by CellTiter 96^®^ AQueous Non-radioactive Cell Proliferation Assay (Promega, Madison, WI).

### qPCR analysis of *soat2* mRNA

Cellular total RNA was extracted and reverse transcribed by random hexamer primers with a RevertAid Reverse Transcriptase (Thermo Fisher Scientific Inc.); the mRNA level of *soat2* was then quantified using the fast SYBR green master mix and the Applied Biosystems StepOnePlus qPCR system (Thermo Fisher Scientific Inc.). The data were normalized to the level of *gapdh* and analysed by the 2^−ΔΔCt^ method. The sequences of primer pairs are shown in Supplementary Table 2.

### SOAT activity assay

The cells at a density of 8 × 10^3^/well in a 96‐well plate with the indicated treatment were incubated in media containing NBD-Chol (1 μg/mL) for 4 h. SOAT activity was evaluated according to the enhanced fluorescence intensity (relative light units, RLU) from the NBD-CE by a reader (Synergy HTX multi-mode, BioTek, Winooski, VT) with excitation at 485 nm and emission at 528 nm after normalization with total protein^51^. For blockage of SOAT activity, the cells were pre-treated with pan inhibitor TMP153 (Cayman Chemical, Ann Arbor, MI) for 16 h before adding NBD-Chol.

### Image tracking of Chol esterification

Huh7 cells were treated with FFAs for 24 h to induce SOAT activity, incubated with NBD-Chol (1 μg/mL) for 30 min, and then visualized with a multi-photon laser scanning microscope (FV1000MPE, Olympus, Tokyo, Japan) for image tracking the NBD-CE by excitation at 485 nm and emission at 528 nm.

### Statistics and reproducibility

The analytic results were expressed as the mean ± SEM. The statistics including Mann–Whitney *U* test for evaluation of differences between groups, and Pearson’s test for correlation were performed with SPSS software version 17.0 (IBM, Armonk, NY). The two-tailed *p* values of <0.05 were considered as a significance.

### Reporting summary

Further information on research design is available in the Nature Research Reporting Summary linked to this article.

## Supplementary information

Supplementary information

Description of Additional Supplementary Files

Supplementary Data 1

Reporting Summary

## Data Availability

The authors declare that the data that support the findings of this study are available from the corresponding author upon reasonable request. The source data underlying Figs. 1b–h,1j, 2a–e, 3a–e, g–l, 4a–c, 5c, d, 6a–g and Supplementary Figs. 1a–c, 2b, 3a, 4a, b, 5a, b, 6a, b, 8a, b, 9a, b, 12a–d, 13a–e, 14a, b are provided as Supplementary Data 1. The IDR region of proteins are predicted under UniProt ID P35610 (SOAT1), O75908 (SOAT2), and P10909 (ApoJ). The newly generated plasmids have been deposited in Addgene, including pSOAT2-DsRed (ID:168276), pΔSOAT2-DsRed (ID:168277), pSOAT1-DsRed (ID:168278), and pΔSOAT1-DsRed (ID:168279).
