# Supplementary material for: Sterol O-acyltransferase 2 chaperoned by apolipoprotein J facilitates hepatic lipid accumulation following viral and nutrient stresses
Source: Commun Biol. 2021 May 12;4:564. doi: 10.1038/s42003-021-02093-2 (PMC8115332; doi:10.1038/s42003-021-02093-2)
Supplement: Supplementary file 1 — Supplementary information [file 42003_2021_2093_MOESM1_ESM.pdf]

Supplementary Information

Supplementary Figure 1. The lipid parameters of wild-type (n=5) and HCV coreTg mice (n=7).

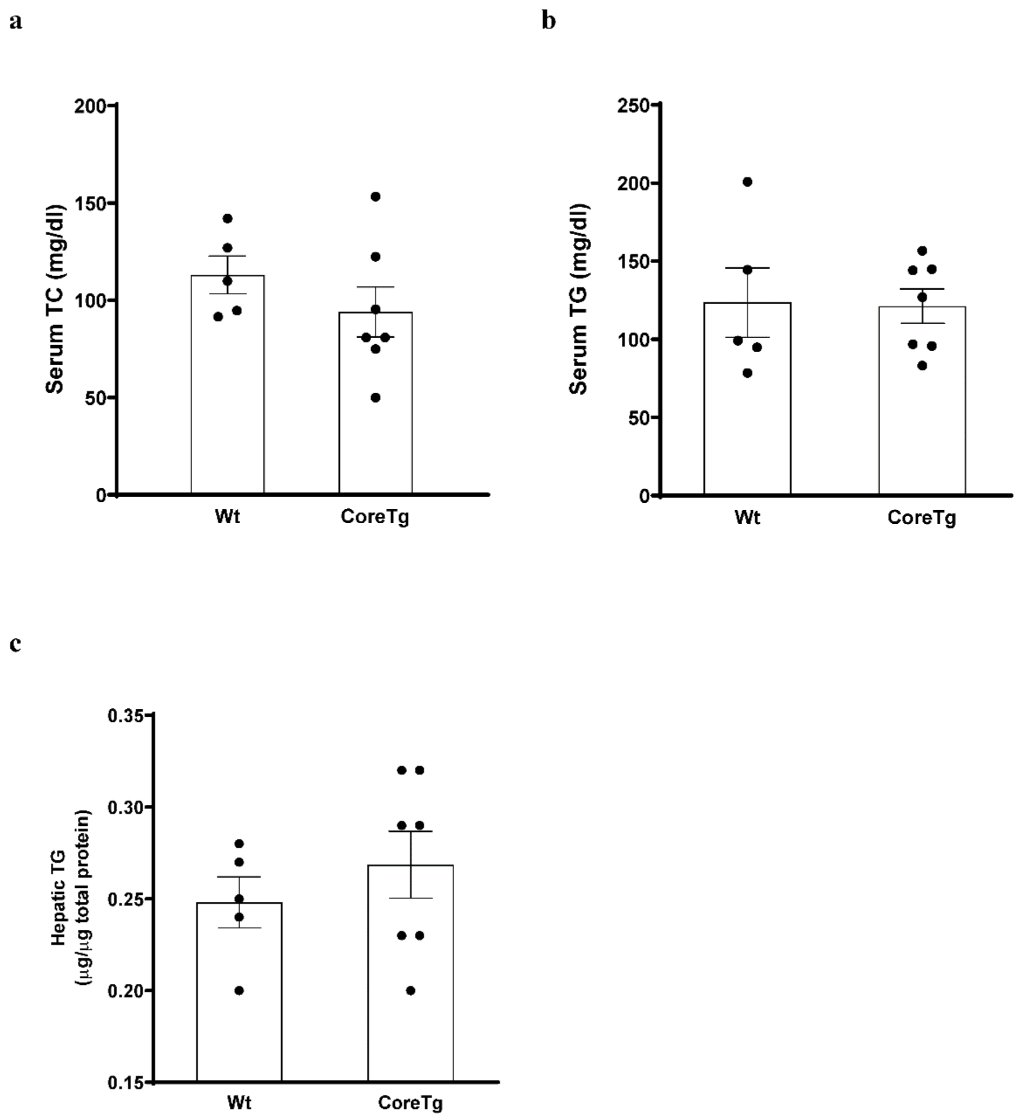

Serum TC (a) and TG (b) were determined as mg/dL. (c) The hepatic TG level was quantified and normalized to the amount of total protein. All the results are presented as the mean±SEM.

Supplementary Figure 2. Knockdown of ApoJ by shRNA in Huh7.5 cells.

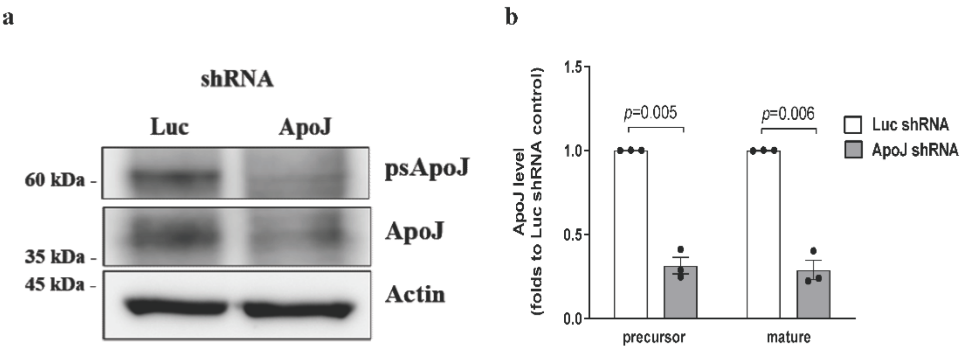

Huh7.5 cells were inoculated with lentivirus bearing Luc shRNA or ApoJ shRNA and subjected to puromycin selection to establish stable cell lines. A representative image of Western blot analysis is shown in the left panel and the intensity of ApoJ in the right panel (n=3). psApoJ; precursor ApoJ.

Supplementary Figure 3. The intracellular lipid contents (a) and HCV core protein level (b) in Huh7.5 cells bearing Luc shRNA or ApoJ shRNA infected with HCV at MOI=0.5.

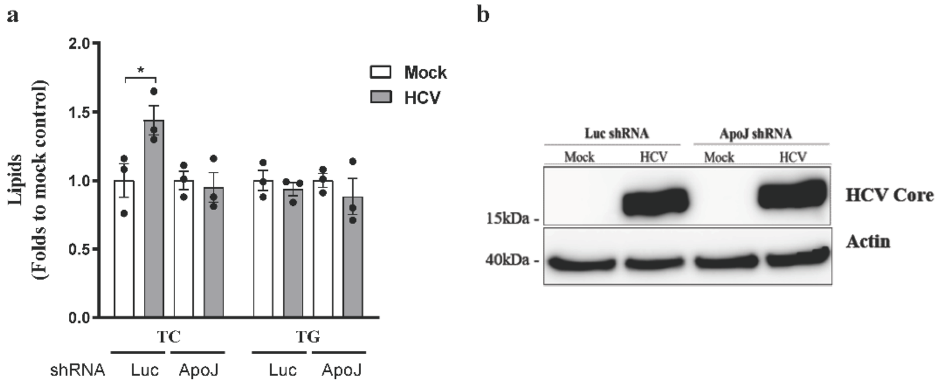

Huh7.5 cells bearing Luc shRNA or ApoJ shRNA were infected with HCV at MOI=0.5 and the lipid contents (a) as well as HCV core protein level (b) were evaluated at day 3 post-infection. The TC and TG levels were normalized with total protein and expressed as folds to mock infection (mean ± SEM, n=3). The expression of HCV core protein was evaluated by Western blot analysis.

Supplementary Figure 4. IFA evaluation of SOAT1/ER (CNX) and SOAT2/ER co-localization with HCV infection.

a

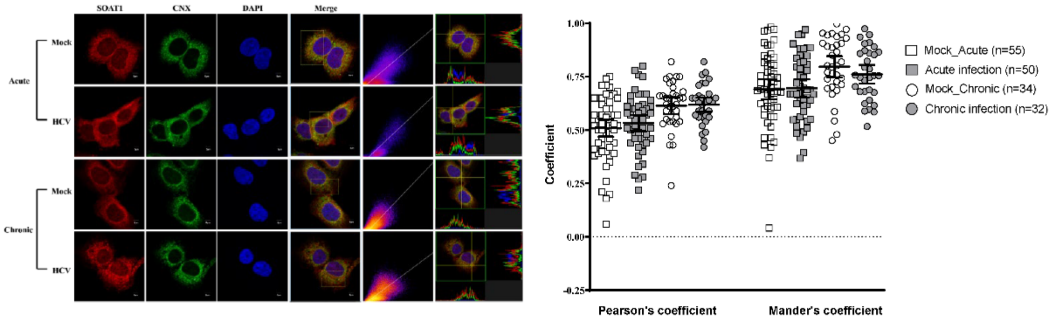

b

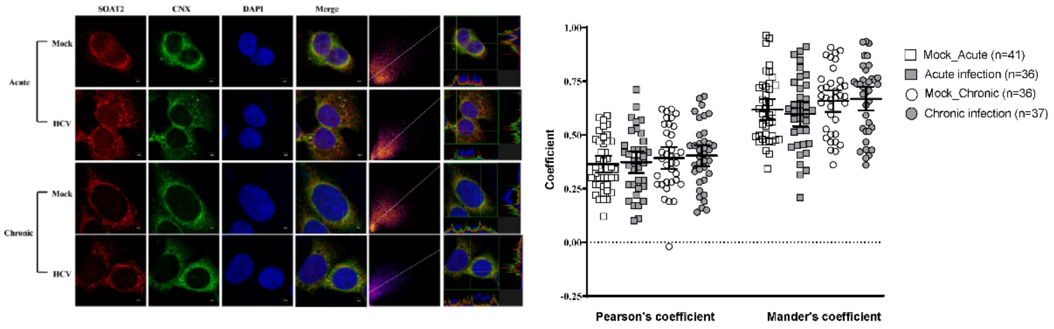

Representative images of immunofluorescence staining of CNX (green) with SOAT1 (red) (a) or with SOAT2 (red) (b) in Huh7.5 cells with acute or chronic HCV infections. The co-localization of SOAT1/CNX and SOAT2/CNX was evaluated as in Fig. 2d. Scale bar, 5 μm.

Supplementary Figure 5. IFA evaluation of SOAT1/Golgi (TGN38) and SOAT1/ApoJ co-localization with HCV infection.

a

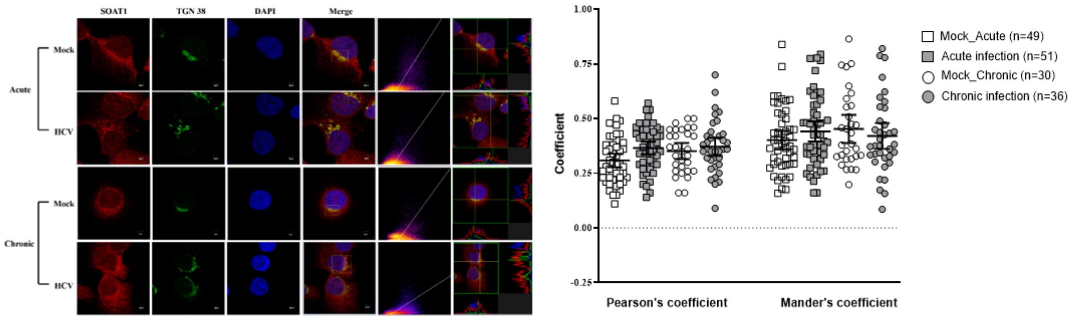

b

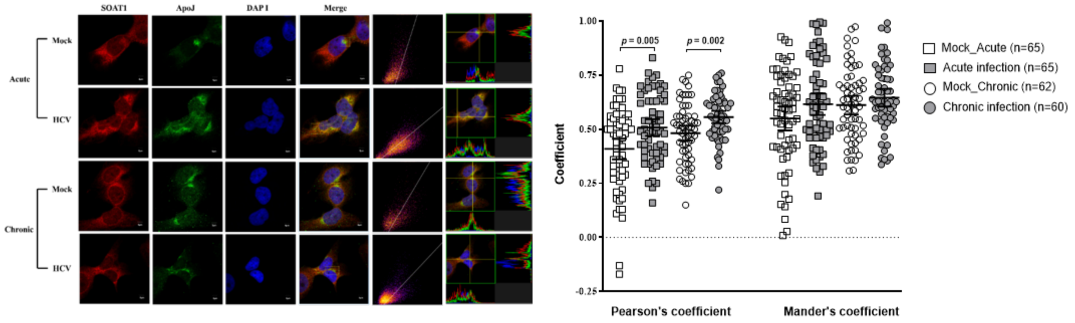

Representative images of immunofluorescence staining of SOAT1 (red) with TGN38 (green) (a) or with ApoJ (green) (b) in Huh7.5 cells with acute or chronic HCV infections. The co-localization of SOAT1/TGN38 and SOAT1/APOJ was evaluated as in Fig. 2d. Scale bar, 5 μm.

Supplementary Figure 6. The minimal effects of FFAs on cell viability.

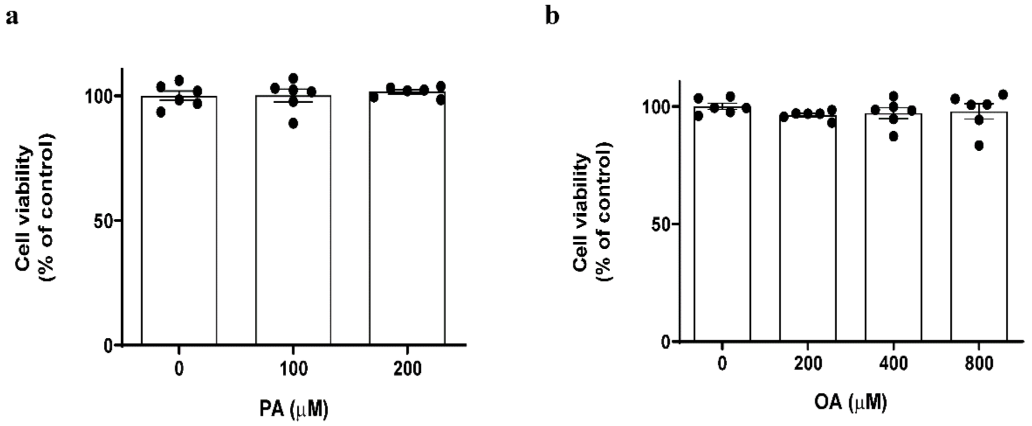

Huh7 cells were exposed to PA (a) and OA (b) at the indicated concentrations for 24 h, and cell viability was determined by the MTS assay. The data are presented as the percentage of alcoholic- and DMSO-treated control cells and expressed as the mean±SEM (n=6).

Supplementary Figure 7. FFAs induced LD accumulation in Huh7 cells.

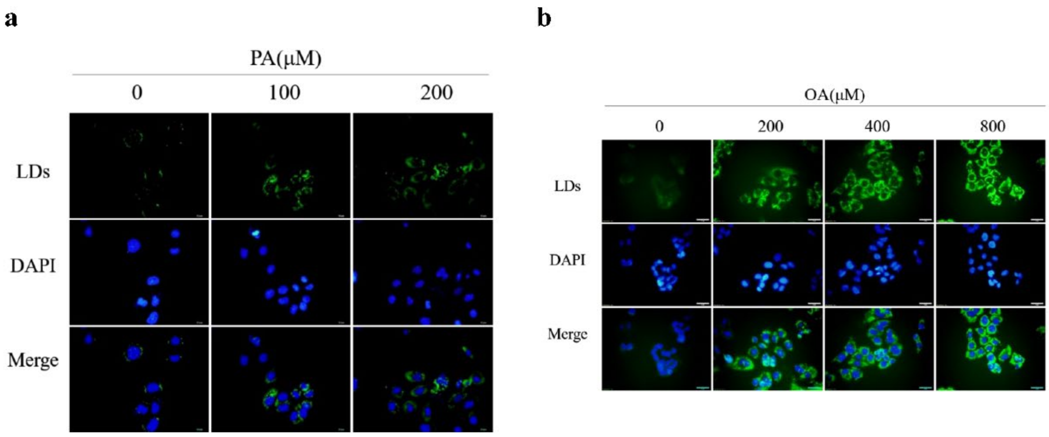

Representative images of LDs in Huh7 cells exposed to sub-lethal doses of PA (a) and OA (b) for 24 h, as shown in Supplementary Fig. 6. The LDs were visualized by BODIPY staining. Scale bar, 20 μm in (a) and 10 μm in (b).

Supplementary Figure 8. IFA evaluation of SOAT1/ER (CNX) and SOAT2/ER co-localization with FFA treatment.

a

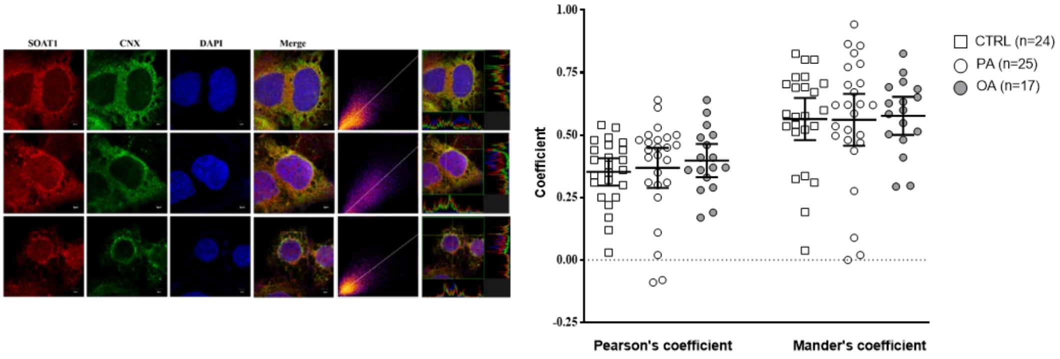

b

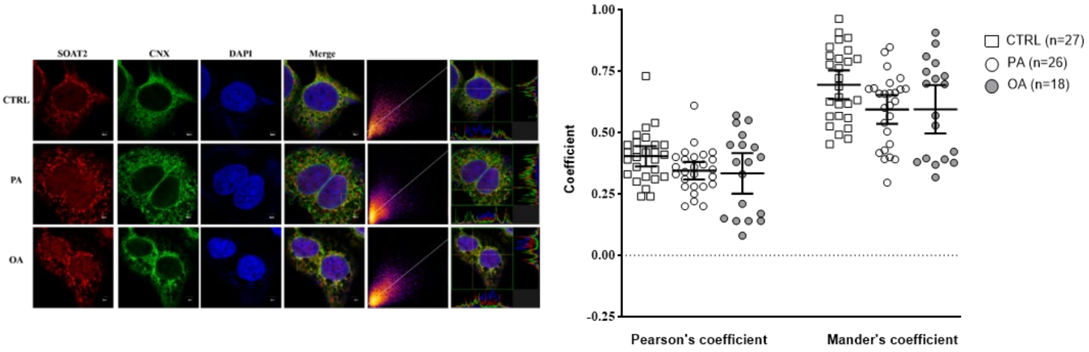

Representative images of immunofluorescence staining of CNX (green) with SOAT1 (red) (a) and with SOAT2 (red) (b) in Huh7 cells treated with FFAs. The co-localization of SOAT1/CNX and SOAT2/CNX was evaluated and presented as in Fig. 2d. Scale bar, 5 μm.

Supplementary Figure 9. FFA treatment had minimal effects on the SOAT1/Golgi (TGN38) and SOAT1/ApoJ co-localization.

a

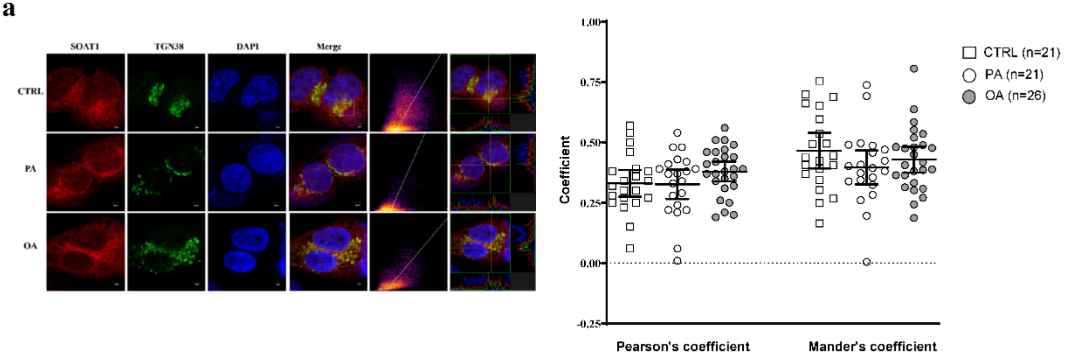

b

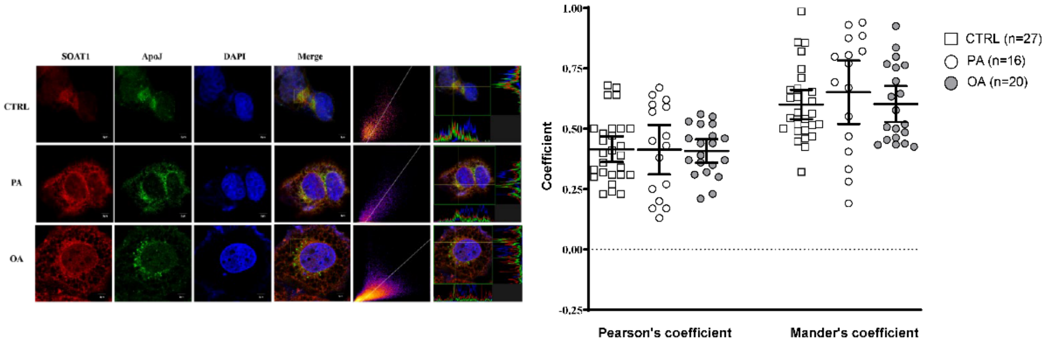

Representative images of immunofluorescence staining of SOAT1 (red) with TGN38 (green) (a) and with ApoJ (green) (b) in Huh7 cells treated with FFAs. Scale bar, 5 µm.

Supplementary Figure 10. Prediction of IDR in ApoJ.

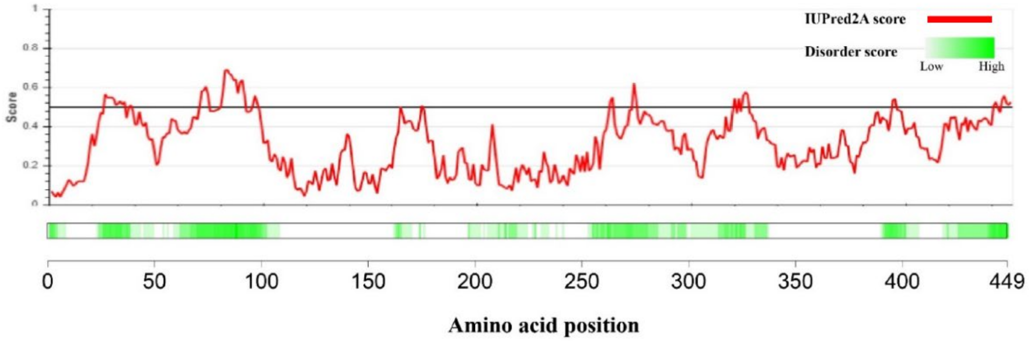

The IDR of ApoJ (UniProt ID: P10909) was predicted by the D^2P^2 and IUPred2A algorithms, respectively.

Supplementary Figure 11. Immunoblotting assay of the recombinant proteins.

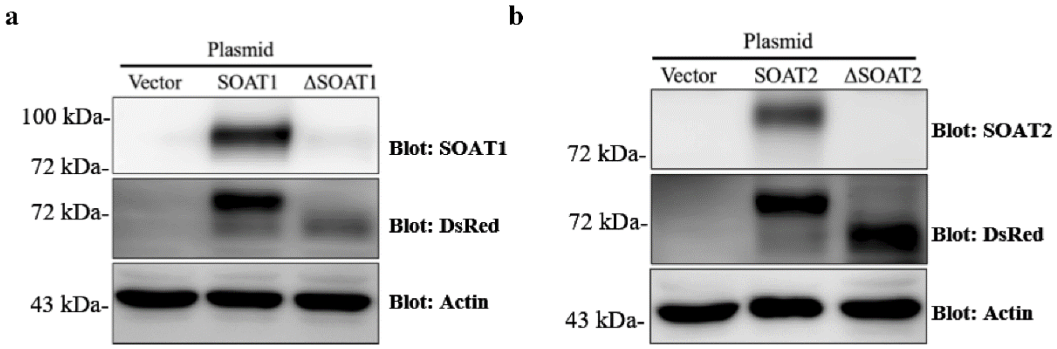

The plasmid constructs containing SOAT1, ΔSOAT1 (a), SOAT2, or ΔSOAT2 (b) were transfected into Huh7 cells and analysed by Western blot for the expression of individual recombinant proteins.

Supplementary Figure 12. The hepatic lipids and ApoJ levels in mice

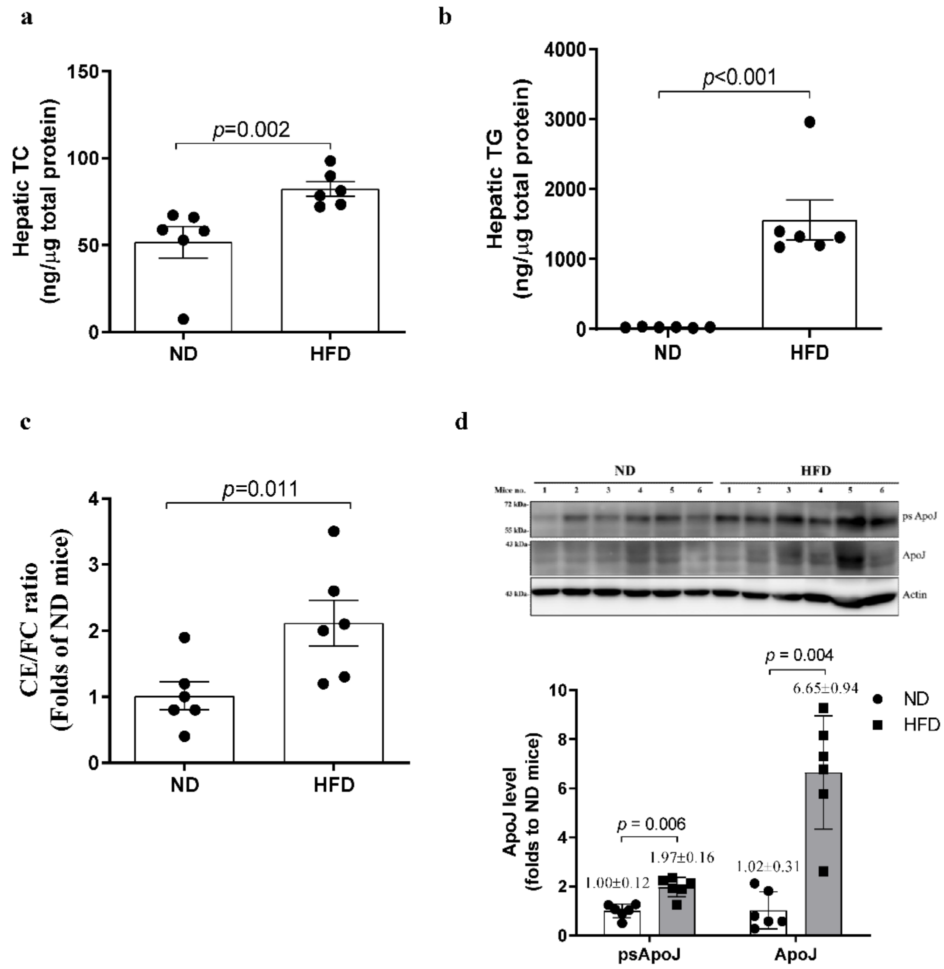

Liver tissues of mice were harvested after the 18-week feeding period with ND or HFD. The hepatic TC (a), TG (b), and CE/FC ratio (c) were determined, normalized to the amount of total protein, and expressed as the mean±range (n=6). (d) The hepatic ApoJ level was determined by Western blot analysis (the left panel). The intensities of psApoJ and ApoJ were determined using AlphaImage software, normalized to actin and expressed as folds to ND mice (mean±SEM, the right panel).

Supplementary Figure 13. The dynamic changes in serum lipid parameters and ApoJ levels.

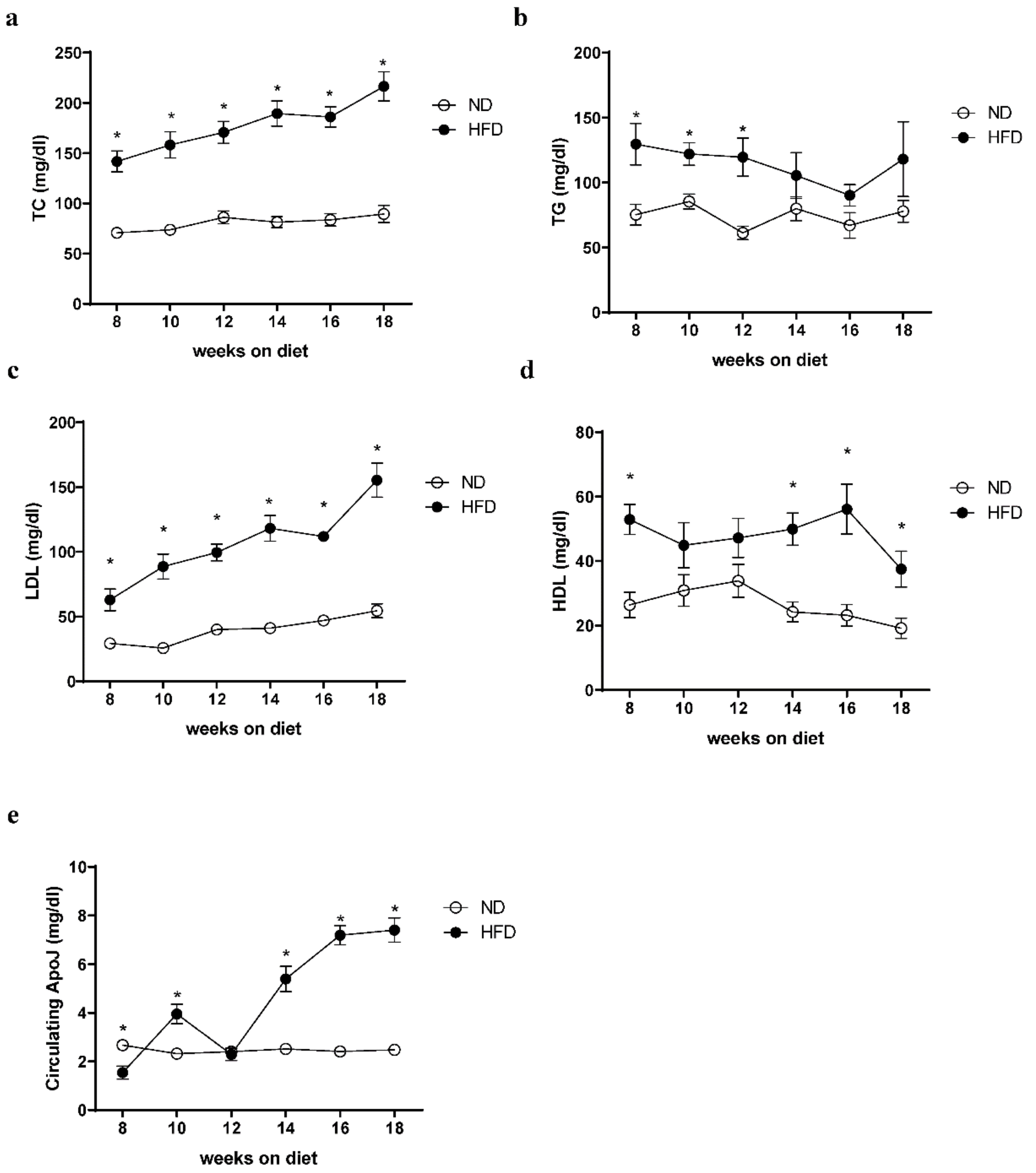

Blood samples were collected from mice at 8, 10, 12, 14, 16, and 18 weeks during feeding period. The levels of TC (a), TG (b), LDL (c), HDL (d), and ApoJ (e) were determined and expressed as the mean±SEM. Asterisks indicate statistical significance ($p<0.05$) between ND and HFD mice.

Supplementary Figure 14. The regression analysis of ApoJ *vs.* TC (a) and LDL (b).

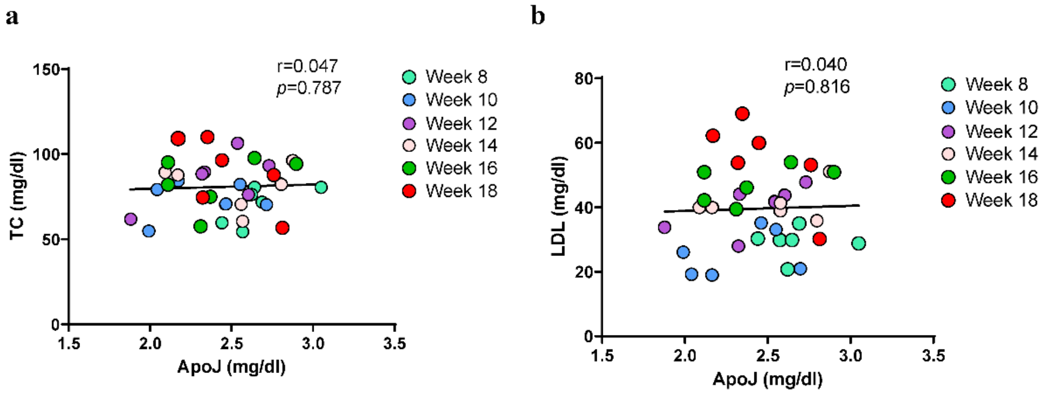

Supplementary Figure 15. Uncropped blots for Figure 1h

	Acute		Chronic		Chronic		
HCV	-	+	-	+	-	+	Control cell
Time (Days)	6	6	123	123	176	176	

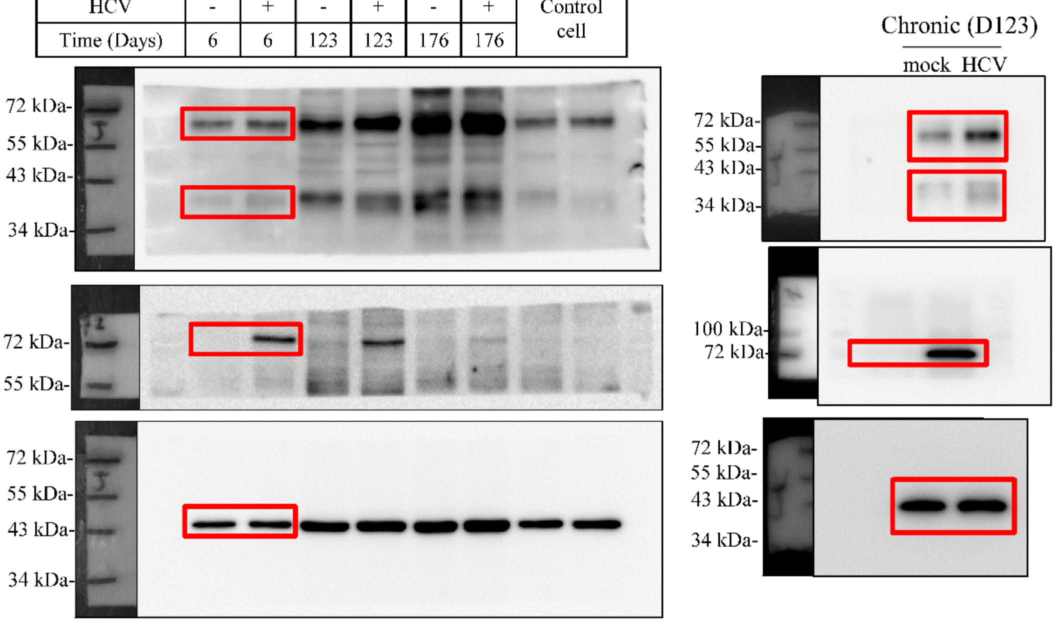

Supplementary Figure 16. Uncropped blots for Figure 1i

Cell line	Huh7.5		Huh7.5		shLuc		shApoJ	
HCV	-	+	-	+	-	+	-	+
Time (Days)	6	6	9	9	6	6	6	6

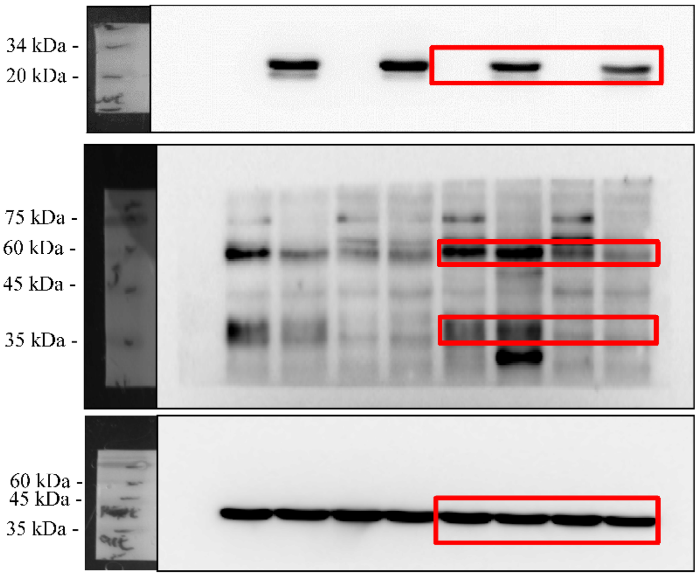

Supplementary Figure 17. Uncropped blots for Figure 2a

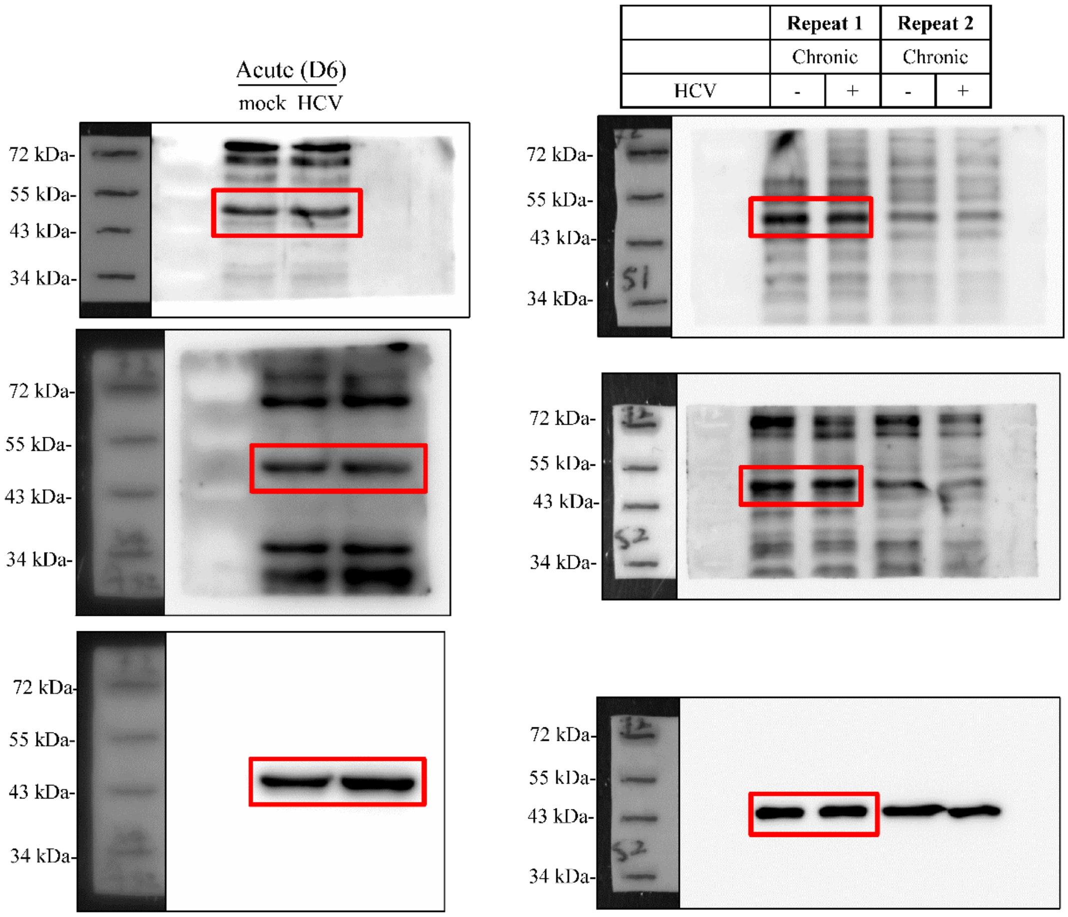

Supplementary Figure 18. Uncropped blots for Figure 3d

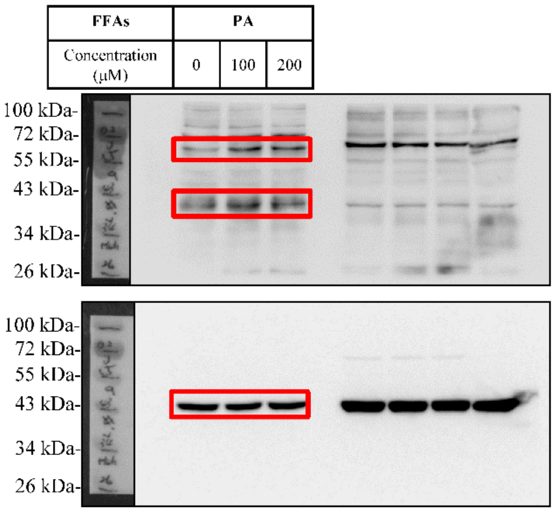

Supplementary Figure 19. Uncropped blots for Figure 3e

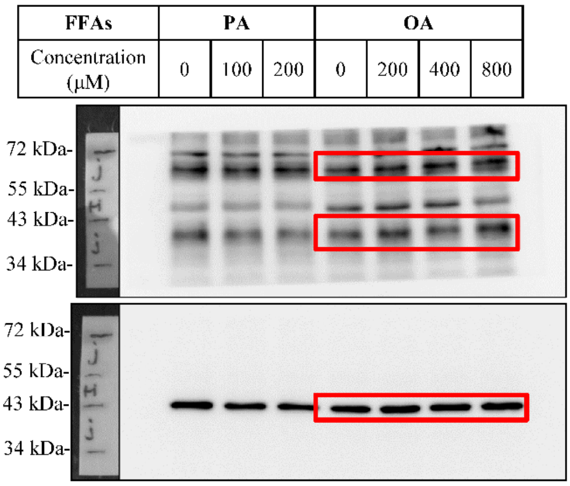

Supplementary Figure 20. Uncropped blots for Figure 3h

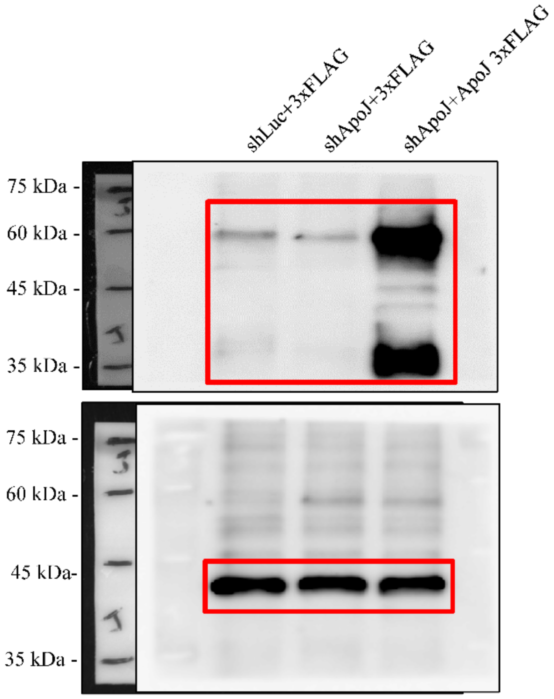

Supplementary Figure 21. Uncropped blots for Figure 3l

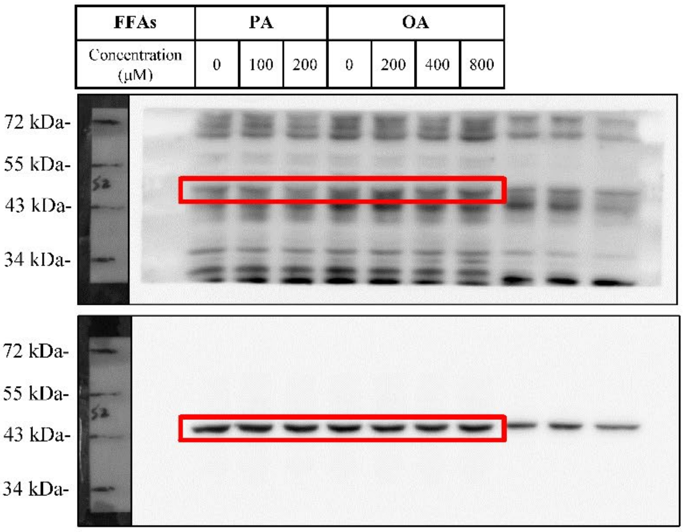

Supplementary Figure 22. Uncropped blots for Figure 3m

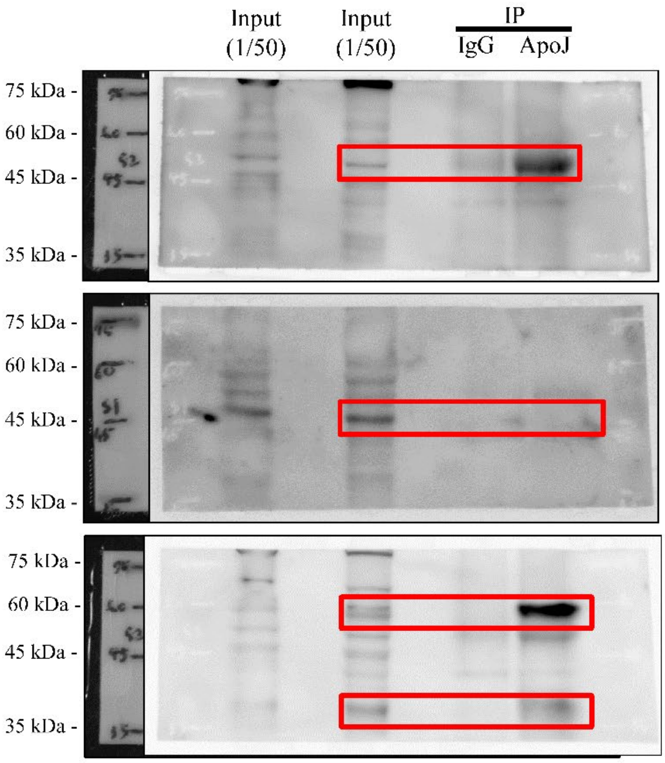

Supplementary Figure 23. Uncropped blots for Figure 4b

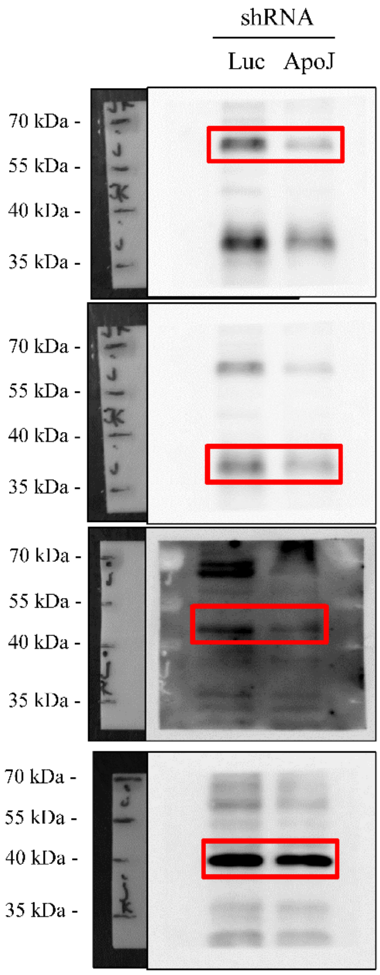

Supplementary Figure 24. Uncropped blots for Figure 5e

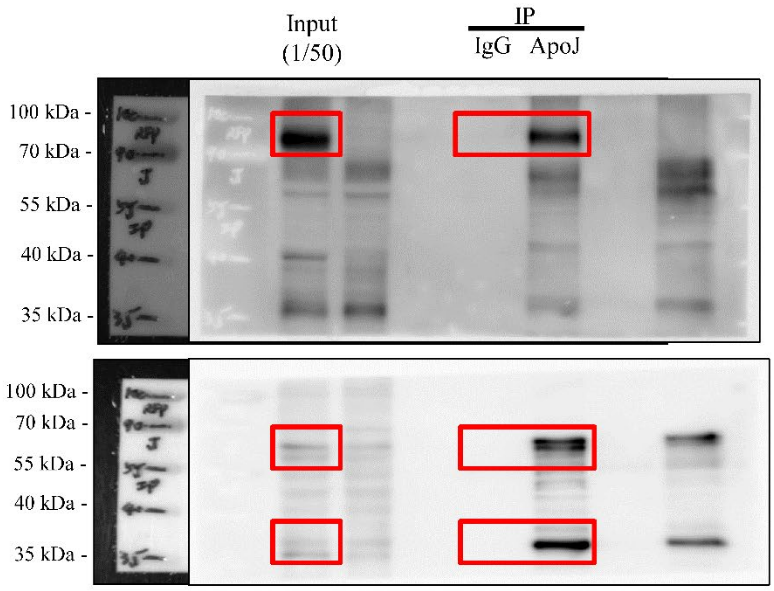

Supplementary Figure 25. Uncropped blots for Figure 5f

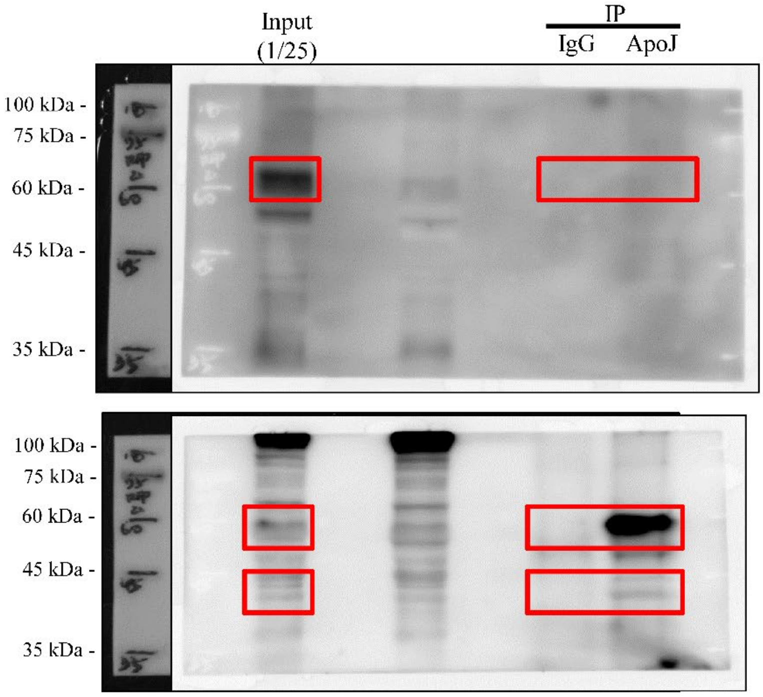

Supplementary Figure 26. Uncropped blots for Supplementary Figure 2a

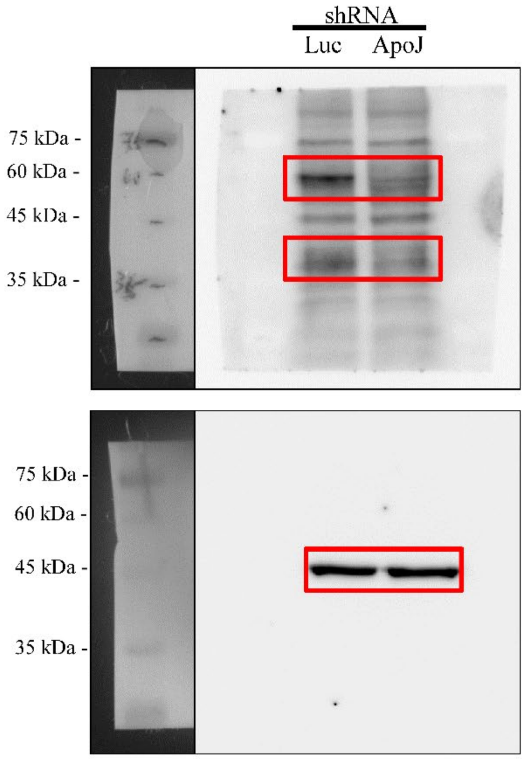

Supplementary Figure 27. Uncropped blots for Supplementary Figure 3b

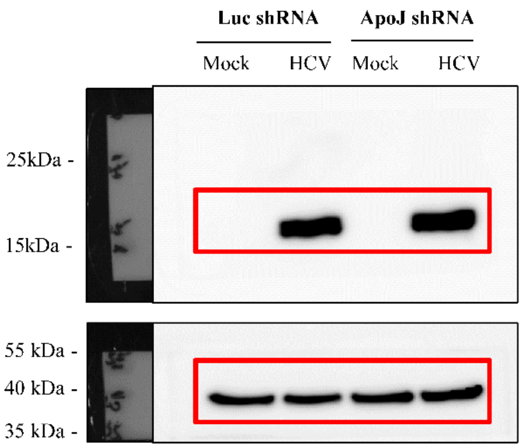

Supplementary Figure 28. Uncropped blots for Supplementary Figure 11a

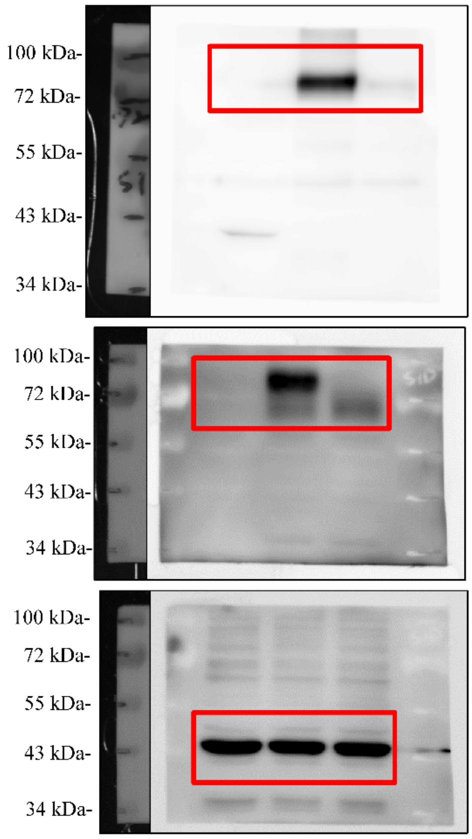

Supplementary Figure 29. Uncropped blots for Supplementary Figure 11b

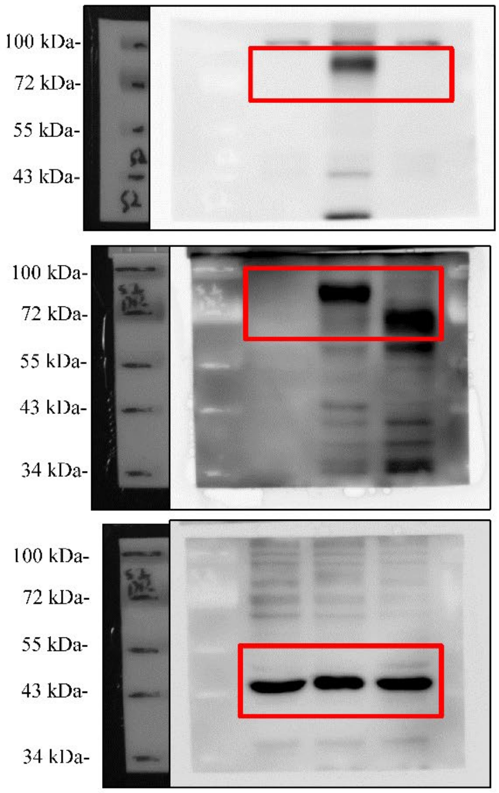

Supplementary Figure 30. Uncropped blots for Supplementary Figure 11d

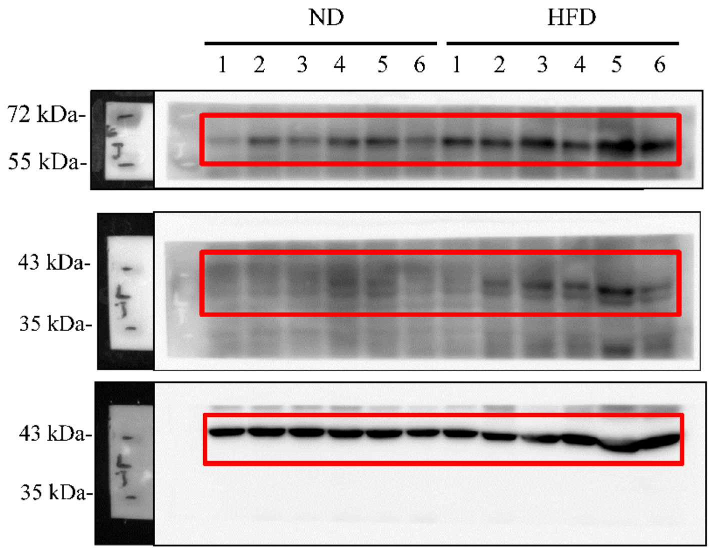

Supplementary Table

Supplementary Table 1. The correlation between circulating ApoJ and serum lipid parameters in mice

	ND		HFD	
	Pearson's r	*p* value	Pearson's r	*p* value
TC (mg/dL)	0.047	0.787	0.566	<0.001
TG (mg/dL)	0.311	0.064	-0.109	0.528
LDL (mg/dL)	-0.019	0.912	0.598	<0.001
HDL (mg/dL)	-0.099	0.565	-0.085	0.624

ND, normal diet; HFD, high fat diet.

Supplementary Table 2. The shRNAs and qPCR primer pairs used in this study

shRNA	
Luciferase (TRCN0000072247)	5'-GAATCGTCGTATGCAGTGAAA-3'
ApoJ (TRCN0000078610)	5'-CAGGGAAGTAAGTACGTCAAT-3'
Primer pairs (forward/reversed) for qPCR analysis	
soat2	5'-CCATGCGGGAGGCTATACAA-3'
	5'-TCATCAAGCAGGGACTTGCG-3'
gapdh	5’-ACCCACTCCTCCACCTTTGAC-3’
	5’-TCCACCACCCTGTTGCTGTAC-3’
