# Supplementary material for: Sterol O-acyltransferase 2 chaperoned by apolipoprotein J facilitates hepatic lipid accumulation following viral and nutrient stresses
Source: Commun Biol. 2021 May 12;4:564. doi: 10.1038/s42003-021-02093-2 (PMC8115332; doi:10.1038/s42003-021-02093-2)
Supplement: Supplementary file 4 — Reporting Summary [file 42003_2021_2093_MOESM4_ESM.pdf]

Corresponding author(s): Kung-Chia Young

Last updated by author(s): Jan 20, 2021

Reporting Summary

Nature Research wishes to improve the reproducibility of the work that we publish. This form provides structure for consistency and transparency in reporting. For further information on Nature Research policies, see our Editorial Policies and the Editorial Policy Checklist.

Statistics

For all statistical analyses, confirm that the following items are present in the figure legend, table legend, main text, or Methods section.

n/a	Confirmed	
☐	☒	The exact sample size (*n*) for each experimental group/condition, given as a discrete number and unit of measurement
☐	☒	A statement on whether measurements were taken from distinct samples or whether the same sample was measured repeatedly
☐	☒	The statistical test(s) used AND whether they are one- or two-sided<br>*Only common tests should be described solely by name; describe more complex techniques in the Methods section.*
☒	☐	A description of all covariates tested
☒	☐	A description of any assumptions or corrections, such as tests of normality and adjustment for multiple comparisons
☐	☒	A full description of the statistical parameters including central tendency (e.g. means) or other basic estimates (e.g. regression coefficient) AND variation (e.g. standard deviation) or associated estimates of uncertainty (e.g. confidence intervals)
☐	☒	For null hypothesis testing, the test statistic (e.g. F, t, r) with confidence intervals, effect sizes, degrees of freedom and P value noted<br>*Give P values as exact values whenever suitable.*
☒	☐	For Bayesian analysis, information on the choice of priors and Markov chain Monte Carlo settings
☒	☐	For hierarchical and complex designs, identification of the appropriate level for tests and full reporting of outcomes
☒	☐	Estimates of effect sizes (e.g. Cohen's d, Pearson's r), indicating how they were calculated

Our web collection on statistics for biologists contains articles on many of the points above.

Software and code

Policy information about availability of computer code

Data collection	The numeric data were collected with Excel or SPSS software version 17.0 (IBM, Armonk, NY), and the image data by the Coloc2 plugin from ImageJ/Fiji (ImageJ-Fiji-ImgLib http://fiji.sc/).
Data analysis	The numeric data were analyzed with SPSS software version 17.0 (IBM, Armonk, NY) and the image data by the Coloc2 plugin from ImageJ/Fiji and FVW31S software.

For manuscripts utilizing custom algorithms or software that are central to the research but not yet described in published literature, software must be made available to editors and reviewers. We strongly encourage code deposition in a community repository (e.g. GitHub). See the Nature Research guidelines for submitting code & software for further information.

Data

Policy information about availability of data

All manuscripts must include a data availability statement. This statement should provide the following information, where applicable:

- Accession codes, unique identifiers, or web links for publicly available datasets
- A list of figures that have associated raw data
- A description of any restrictions on data availability

The authors declare that the data that support the findings of this study are available from the corresponding author upon reasonable request. The source data underlying Figs. 1B-H, 1J, 2A-E, 3A-E, G-L, 4A-C, 5C, D, 6A-G and Supplementary Figs. 1A-C, 2B, 3A, 4A, B, 5A, B, 6A, B, 8A, B, 9A, B, 12A-D, 13A-E, 14A, B are provided as Source Data file.

Field-specific reporting

Please select the one below that is the best fit for your research. If you are not sure, read the appropriate sections before making your selection.

☒ Life sciences ☐ Behavioural & social sciences ☐ Ecological, evolutionary & environmental sciences

For a reference copy of the document with all sections, see nature.com/documents/nr-reporting-summary-flat.pdf

Life sciences study design

All studies must disclose on these points even when the disclosure is negative.

Sample size	For image analysis within the successfully replicated experiments, the positive cells with consistently stained known markers were chosen to constitute the sample size. Moreover, the staining patterns between cells should be observed with low variability as shown in each figure. For correlation study with human participants n>30 and animal nĩ 6 for each group, the sample sizes were determined based on our previous experience on patient recruitment.
Data exclusions	Data were not excluded from analysis
Replication	For each quantitative assays and WB, biological and technical repeats for at least n=3 were applied. Immunoprecipitation assays were successfully duplicated.
Randomization	Mice were randomized as high-fat diet experimental group and normal diet control group.
Blinding	No blinding was applied to the investigators.

Reporting for specific materials, systems and methods

We require information from authors about some types of materials, experimental systems and methods used in many studies. Here, indicate whether each material, system or method listed is relevant to your study. If you are not sure if a list item applies to your research, read the appropriate section before selecting a response.

Materials & experimental systems

n/a	Involved in the study	
☐	☒	Antibodies
☐	☒	Eukaryotic cell lines
☒	☐	Palaeontology and archaeology
☐	☒	Animals and other organisms
☐	☒	Human research participants
☒	☐	Clinical data
☒	☐	Dual use research of concern

Methods

n/a	Involved in the study	
☒	☐	ChIP-seq
☒	☐	Flow cytometry
☒	☐	MRI-based neuroimaging

Antibodies

Antibodies used	Monoclonal antibodies recognizing HCV core (ab2740) and NS3 (ab65407) were purchased from Abcam (Cambridge, UK); ApoJ (ARG62961) for IFA from Arigo Biolaboratories (Taipei, Taiwan); actin (MAB1501) from Millipore (Billerica, MA); and DsRed (tcba13674) from Taiclone Biotech Corp. (Taipei, Taiwan). Polyclonal antibodies recognizing human ApoJ for WB analysis (ab69644) was purchased from Abcam; human ApoJ (sc-6419) for immunoprecipitation from Santa Cruz Biotechnology (Santa Cruz, CA); mouse ApoJ for WB analysis (PA5-46931) from Thermo Fisher Scientific Inc. (Waltham, MA); SOAT1 (ARG56476) and SOAT2 (ARG57814) for WB analysis from Arigo Biolaboratories; and SOAT 1 (bs-7544R) and SOAT 2 (bs-5020R) for IFA from Bioss Antibodies (Beijing, China). Goat anti-mouse Alexa-488-, and anti-rabbit Alexa-568-conjugated secondary antibodies were purchased from Thermo Fisher Scientific Inc; Goat anti-mouse HRP- and anti-rabbit HRP-conjugated secondary antibodies were purchased from Chamot Biotechnology CO. LTD. (Shanghai, China).
Validation	All antidodies were obtained commercially and the validation of antibodies were available in datasheet provided by corresponding supplyers.

Eukaryotic cell lines

Policy information about cell lines

Cell line source(s)	Huh7 cells were purchased form ATCC.<br>Huh7.5 cells were provided by Professor Charles M. Rice, Rockefeller University.

Authentication	Huh7 cells were authenticated by STR analysis and morphology; Huh7.5 cells by morphology and susceptibility to HCV infection.
Mycoplasma contamination	The cells were maintained routinely in culture medium containing mycoplasma inhibitor to prevent and eliminate mycoplasma contamination.
Commonly misidentified lines (See ICLAC register)	None

Animals and other organisms

Policy information about studies involving animals; ARRIVE guidelines recommended for reporting animal research

Laboratory animals	Six-week-old male C57BL/6 mice were used.
Wild animals	None
Field-collected samples	None
Ethics oversight	This study was approved by the local Animal Research Committee of NCKU, and all experimental procedures followed the guidelines of the Public Health Service policy on Humane Care and Use of Laboratory Animals. The information about ethics has been provided in Materials and Methods section of the manuscript.

Note that full information on the approval of the study protocol must also be provided in the manuscript.

Human research participants

Policy information about studies involving human research participants

Population characteristics	Human participants were 36 chronic hepatitis C patients (24 female and 12 male; aged from 46-81 years old) with normal haemoglobin A1c (< 6%), and in the absence of other forms of viral hepatitis and liver diseases, and 41 non-alcoholic fatty liver disease (17 female and 24 male; aged from 25-85 years old) with normal haemoglobin A1c (< 6%), and in the absence of alcohol consumption (<20 g/day), hepatitis B or C virus infections and other forms of liver diseases. Diagnosis were based on the clinical examinations, laboratory data and physician interpretations.
Recruitment	Human participants were recruited from 2017 to 2020 in the physician clinics of Tainan Municipal Hospital, Taiwan. The venous blood were collected, centrifuged and the serum samples stored at -80°C until analysis.
Ethics oversight	The study was approved by the local Institutional Review Board of Tainan Municipal Hospital and each participant signed an informed consent form. The information about ethics has been provided in Materials and Methods section of the manuscript.

Note that full information on the approval of the study protocol must also be provided in the manuscript.
